# Deep learning models incorporating endogenous factors beyond DNA sequences improve the prediction accuracy of base editing outcomes

**DOI:** 10.1038/s41421-023-00624-1

**Published:** 2024-02-20

**Authors:** Tanglong Yuan, Leilei Wu, Shiyan Li, Jitan Zheng, Nana Li, Xiao Xiao, Haihang Zhang, Tianyi Fei, Long Xie, Zhenrui Zuo, Di Li, Pinzheng Huang, Hu Feng, Yaqi Cao, Nana Yan, Xinming Wei, Lei Shi, Yongsen Sun, Wu Wei, Yidi Sun, Erwei Zuo

**Affiliations:** 1 Shenzhen Branch, Guangdong Laboratory for Lingnan Modern Agriculture, Key Laboratory of Synthetic Biology, Ministry of Agriculture and Rural Affairs, Agricultural Genomics Institute at Shenzhen, Chinese Academy of Agricultural Sciences, Shenzhen, Guangdong China; 2 Institute of Neuroscience, CAS Center for Excellence in Brain Science and Intelligence Technology, Chinese Academy of Sciences, Shanghai, China; 3 Bio-Med Big Data Center, Key Laboratory of Computational Biology, Shanghai Institute of Nutrition and Health, University of Chinese Academy of Sciences, Chinese Academy of Sciences, Shanghai, China; 4 State Key Laboratory for Conservation and Utilization of Subtropical Agro-Bioresources, Guangxi University, Nanning, Guangxi China; 5 Key Laboratory of Agricultural Animal Genetics, Breeding and Reproduction of Ministry of Education & Key Lab of Swine Genetics and Breeding of Ministry of Agriculture and Rural Affairs, Huazhong Agricultural University, Wuhan, Hubei China; 6 Epigenic Therapeutics, Inc., Shanghai, China; 7 Lingang Laboratory, Shanghai, China

**Keywords:** Bioinformatics, Cell biology

## Abstract

Adenine base editors (ABEs) and cytosine base editors (CBEs) enable the single nucleotide editing of targeted DNA sites avoiding generation of double strand breaks, however, the genomic features that influence the outcomes of base editing in vivo still remain to be characterized. High-throughput datasets from lentiviral integrated libraries were used to investigate the sequence features affecting base editing outcomes, but the effects of endogenous factors beyond the DNA sequences are still largely unknown. Here the base editing outcomes of ABE and CBE were evaluated in mammalian cells for 5012 endogenous genomic sites and 11,868 genome-integrated target sequences, with 4654 genomic sites sharing the same target sequences. The comparative analyses revealed that the editing outcomes of ABE and CBE at endogenous sites were substantially different from those obtained using genome-integrated sequences. We found that the base editing efficiency at endogenous target sites of both ABE and CBE was influenced by endogenous factors, including epigenetic modifications and transcriptional activity. A deep-learning algorithm referred as BE_Endo, was developed based on the endogenous factors and sequence information from our genomic datasets, and it yielded unprecedented accuracy in predicting the base editing outcomes. These findings along with the developed computational algorithms may facilitate future application of BEs for scientific research and clinical gene therapy.

## Introduction

Single nucleotide variants (SNVs) represent more than half of pathogenic mutations in the human genome, and an accurate reversion of SNVs is one of the most important goals for gene therapy^1^. Base editors (BEs), including ABEs^2^ and CBEs^3^, have been widely used to correct pathogenic point mutations^4–7^ and to generate animal disease models^8^. However, experimental evaluation of editing outcomes is time-consuming, and this limits its application to only a small number of target sites^9,10^. Several computational methods have recently been developed to predict the editing outcomes of BEs using targeted sequence information from lentiviral integrated libraries in mammalian cells^9,11–14^. A lentiviral integrated library usually comprises thousands of oligonucleotides, each of which encodes a unique 20 nucleotide (nt) small guide RNA (sgRNA) spacer with paired target sequence. The sgRNA library is randomly integrated into the genome of mammalian cells, and the sgRNAs could express under the drive of human U6 promoter. The expressed sgRNA combines with transfected or genome-integrated BEs to induce base editing at the integrated target sequence. Then the integrated paired target sequences were PCR amplified and subjected to sequencing for measuring editing efficiency^9,11–14^. Previous studies demonstrated that endogenous factors, such as transcriptional activity and chromatin accessibility, were strictly connected with the cleavage efficiency of CRISPR-Cas9 endonuclease^15–19^. The lentiviral integrated libraries limit the examination of endogenous factors at target sites, given the target sequences were randomly integrated into the genome. Therefore, large genome-wide endogenous datasets should be generated to elucidate the influence of endogenous factors on base editing. Computational methods incorporating endogenous factors of great importance could then be developed for better predicting the outcomes of endogenous base editing.

In this study, we performed base editing experiments of ABE and CBE on a large number of endogenous genomic sites and on a lentiviral integrated library in human embryonic kidney (HEK) 293 T cells. The comparative analyses performed in this study showed that the editing outcomes at the endogenous sites were greatly influenced by endogenous factors, including transcriptional activity and epigenetic factors, such as chromatin accessibility, DNA and histone modifications, genome-associated protein factors, and cis-regulatory elements (CREs)^20–23^. A deep-learning algorithm for an accurate prediction of the editing outcomes of BEs was developed by incorporating information of endogenous factors and DNA sequences. These findings are beneficial to understand which are the factors that may contribute the most to the base editing outcomes and provide a computational tool for an optimal sgRNA selection for future base editing applications.

## Results

### Generation of genome-wide endogenous and lentiviral-integrated base editing datasets

**Fig. 1:**
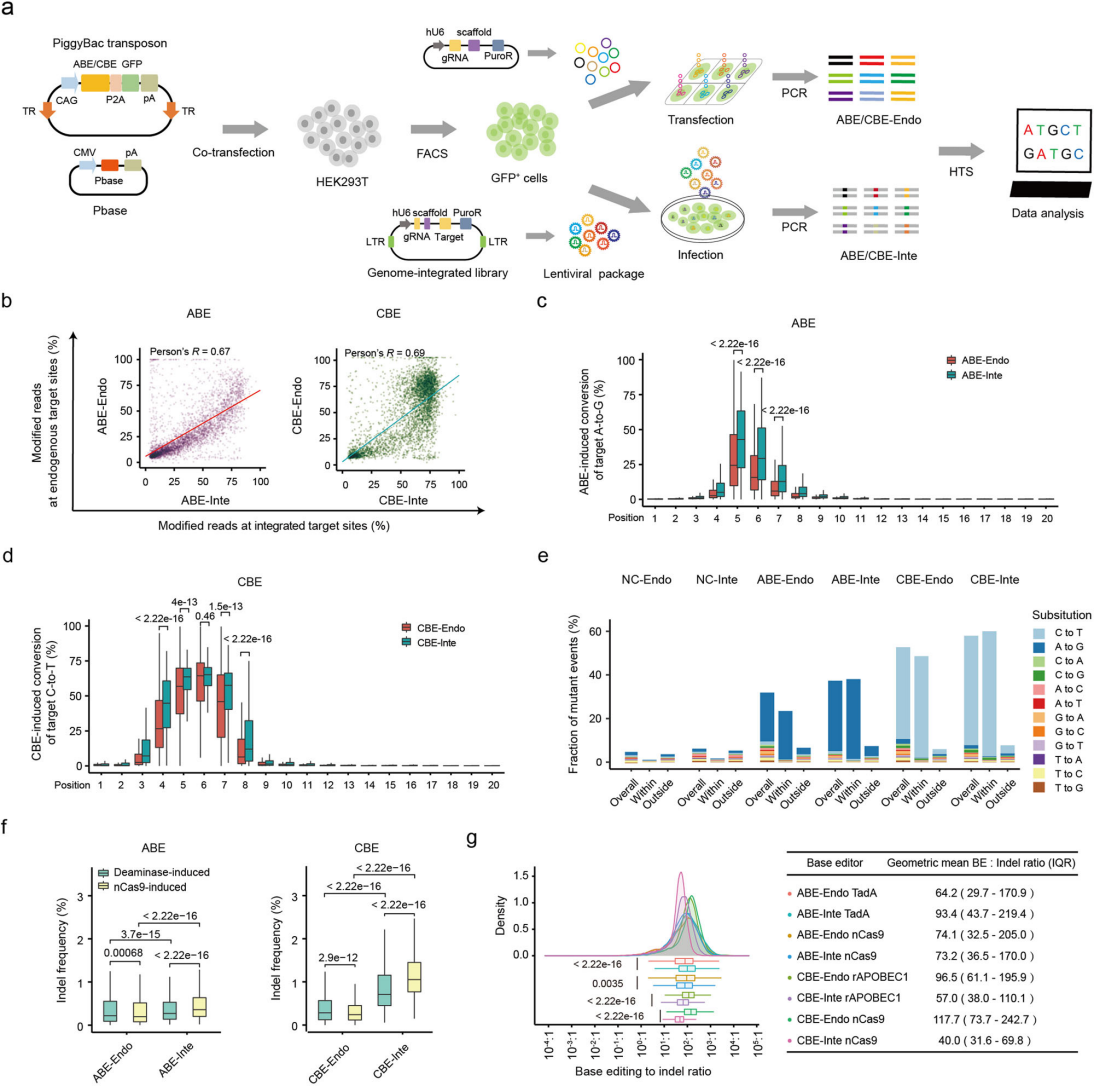
Systematic characterization of base editing activity at endogenous and genome-integrated target sites. **a** Overview of the generation of genome-wide endogenous and integrated ABE and CBE datasets. BEs expressing cassettes were stably integrated into HEK293T cells, followed by infection with paired lentiviral sgRNA library or transfected with one of the sgRNA expressing vectors. The successfully infected or transfected cells were enriched by antibiotic selection, and then PCR-amplification and next generation sequencing were performed for the target sequences. **b** Correlation of ABE- or CBE-directed editing efficiencies between endogenous and integrated target sites with the same target sequences. *n* = 3987 for ABE; *n* = 4001 for CBE. **c**, **d** A-to-G (**c**) and C-to-T (**d**) base editing efficiencies at each protospacer positions 1–20 (PAM is at positions 21–23) of endogenous and integrated target sites. Data are presented as mean values ± SEM. *n* = 718 (position 1), 1245, 1607, 809, 1343, 1250, 1106, 916, 916, 1157, 1269, 1493, 898, 1109, 1300, 1234, 936, 771, 910 and 1008 (position 20) for ABE. *n* = 576 (position 1), 1060, 563, 1025, 1181, 1040, 1195, 1343, 1082, 1079, 1065, 975, 1357, 1163, 938, 1156, 1136, 1891, 931 and 572 (position 20) for CBE. **e** Proportions of 12 base conversion types within and outside of editing windows (5–7 for ABE; 4–8 for CBE) in the Endo- and Inte- datasets. **f** Box plot depicting the deaminase (rAPOBEC1 or TadA) or nCas9-directed indel frequencies in the indicated datasets. *n* = 3938 (ABE-Endo TadA), 3904 (ABE-Inte TadA), 3970 (CBE-Endo rAPOBEC1), 3991 (CBE-Inte rAPOBEC1), 3958 (ABE-Endo nCas9), 3827 (ABE-Inte nCas9), 3974 (CBE-Endo nCas9) and 3972 (CBE-Inte nCas9). **g** Base editing: indel ratio distributions for deaminase and nCas9-induced indels at endogenous and integrated target sites. *n* = 2910 (ABE-Endo TadA), 2819 (ABE-Inte TadA), 3464 (CBE-Endo rAPOBEC1), 3450 (CBE-Inte rAPOBEC1), 2899 (ABE-Endo nCas9), 2879 (ABE-Inte nCas9), 3461 (CBE-Endo nCas9) and 3463 (CBE-Inte nCas9).

To explore the factors that could contribute to the efficiency of the base editing at endogenous target sites, we generated genome-wide datasets for two high-fidelity BEs: ABEmax^F148A^ (F148A mutation in TadA, referred as ABE)^24^ and YE1-BE3-FNLS (W90Y and R126E mutations in rAPOBEC1, referred as CBE)^25^ (Fig. 1a; Supplementary Fig. S1a). Specifically, expression cassettes harboring ABE or CBE were stably integrated into the genome of human HEK293T cells using the PiggyBac transposon system (Fig. 1a; Supplementary Fig. S1a, b). More in detail, a total of 5012 sgRNAs targeting 4262 (randomly selected) genes across the genome were designed (Supplementary Fig. S1c, d and Table S1). Each 20-base pair (bp) sgRNA spacer was cloned into the vector pLenti-guide-puro containing an upstream U6 promoter, followed by the transfection into HEK293T cells that stably expressed ABE or CBE (Fig. 1a; Supplementary Fig. S1a). In addition, a lentiviral integrated library of 11,868 sgRNA oligonucleotides, 4654 of which were shared with the endogenous sites, was developed. Each oligonucleotide encoded a unique 20 nt sgRNA spacer paired with a target sequence (Supplementary Table S1). Then, the BEs stably expressing HEK293T cells were infected with the lentiviral sgRNA library at Multiplicity of Infection (MOI) = 0.3 (Fig. 1a). Genomic DNAs were then extracted from transfected or infected cells in order to perform a specific PCR followed by high-throughput sequencing. To ensure the robustness of the editing outcomes, three independently transfected and two independently infected replicates were generated for endogenous and integrated datasets, respectively.

Using a coverage of > 100×, sequencing data were generated for a total of 4529 endogenous and 11,112 integrated sites for ABE (referred as “ABE-Endo” and “ABE-Inte”, respectively), and for 4587 endogenous and 11,002 integrated sites for CBE (referred as “CBE-Endo” and “CBE-Inte”, respectively) (Supplementary Fig. S1e). The endogenous and integrated datasets shared 3987 and 4001 sites with the same target sequences for ABE and CBE, respectively (Supplementary Fig. S1e). A high reproducibility of the editing outcomes between replicates in each dataset was observed (*R* = 0.95–0.99; Supplementary Fig. S1f–h), while the comparison of the editing efficiencies at the target sites revealed only a modest correlation between endogenous and integrated datasets for ABE (*R* = 0.67) or CBE (*R* = 0.69) (Fig. 1b). The editing windows for ABE- and CBE-directed base editing was located at positions 5–7 and 4–8 within the endogenous datasets, consistent with the results obtained in the integrated datasets and in previous reports^24,25^ (Fig. 1c, d). The C-to-T editing efficiency values at positions 5 and 6 ranged from 0% to 100% in CBE-Endo dataset, but from 25% to 75% in the integrated dataset (Fig. 1d). Despite the high product purity in all 4 datasets, the average efficiency of the desired editing (A-to-G or C-to-T) in Endo-datasets was much lower than that observed in Inte-datasets (Fig. 1e). Additionally, the frequency of bystander edits was more elevated within and outside the editing window in the Endo-datasets than in the Inte-datasets for both ABE and CBE (Supplementary Fig. S1i). All together, these data, including the low editing efficiency and increased complexity of the editing products at endogenous target sites, suggested the necessity to investigate the main determinants of base editing outcomes using genome-wide endogenous datasets.

### Characterization of indels induced by BEs

In addition to the differences in base editing efficiencies, we also observed lower indel frequency values in Endo-datasets, as compared to Inte-datasets (Fig. 1f; Supplementary Fig. S2a). Indeed, the highest frequency of 1 bp insertion was detected at position 18 of the protospacer (PAM as positions 21–23) in all 4 datasets, caused by the activity of Cas9 (nCas9, D10A) HNH nuclease domain^26^ (Supplementary Fig. S2b). Consistent with their editing windows, ABE and CBE induced 1 bp deletions at positions 5–7 and 4–9 at a high frequency in both Endo- and Inte-datasets (Supplementary Fig. S2b). These indels occurring within the editing windows were induced by base excision repair following the deaminase TadA- or rAPOBEC1-induced deamination^27,28^. The indel frequency induced by deaminase (positions 1–11) was higher in Endo-datasets but lower in Inte-datasets compared to that induced by nCas9 (positions 14–20) (Fig. 1f), while the frequency of deaminase-induced indels increased with the number of target Cs for CBE at both endogenous and integrated sites, but not with the target A numbers for ABE (Supplementary Fig. S2c, d), suggesting that rAPOBEC1, but not TadA, has a higher deaminase activity at continuous targeted bases. Additionally, high ratios of base editing efficiency to indel frequency in ABE-Endo and CBE-Endo datasets were observed, consistently with what observed in the integrated datasets (Fig. 1g; Supplementary Fig. S2a) and in a previous study^11^. This value was substantially higher in CBE-Endo dataset than CBE-Inte dataset for both deaminase rAPOBEC1 (geometric mean: 96.5 vs 57.0) and nCas9-induced indels (117.7 vs 40.0), while it was lower in ABE-Endo dataset than ABE-Inte dataset for deaminase TadA-induced indels (64.2 vs 93.4) but similar for nCas9-induced indels (74.1 vs 73.2) (Fig. 1g). Moreover, the indel frequency induced by both nCas9 and deaminase activity increased with the base editing efficiency of BEs in the Inte-datasets. On the contrary, no statistical association was observed for the Endo-datasets (Supplementary Fig. S2e, f).

### Characterization of the effects of sequence features on base editing efficiency

**Fig. 2:**
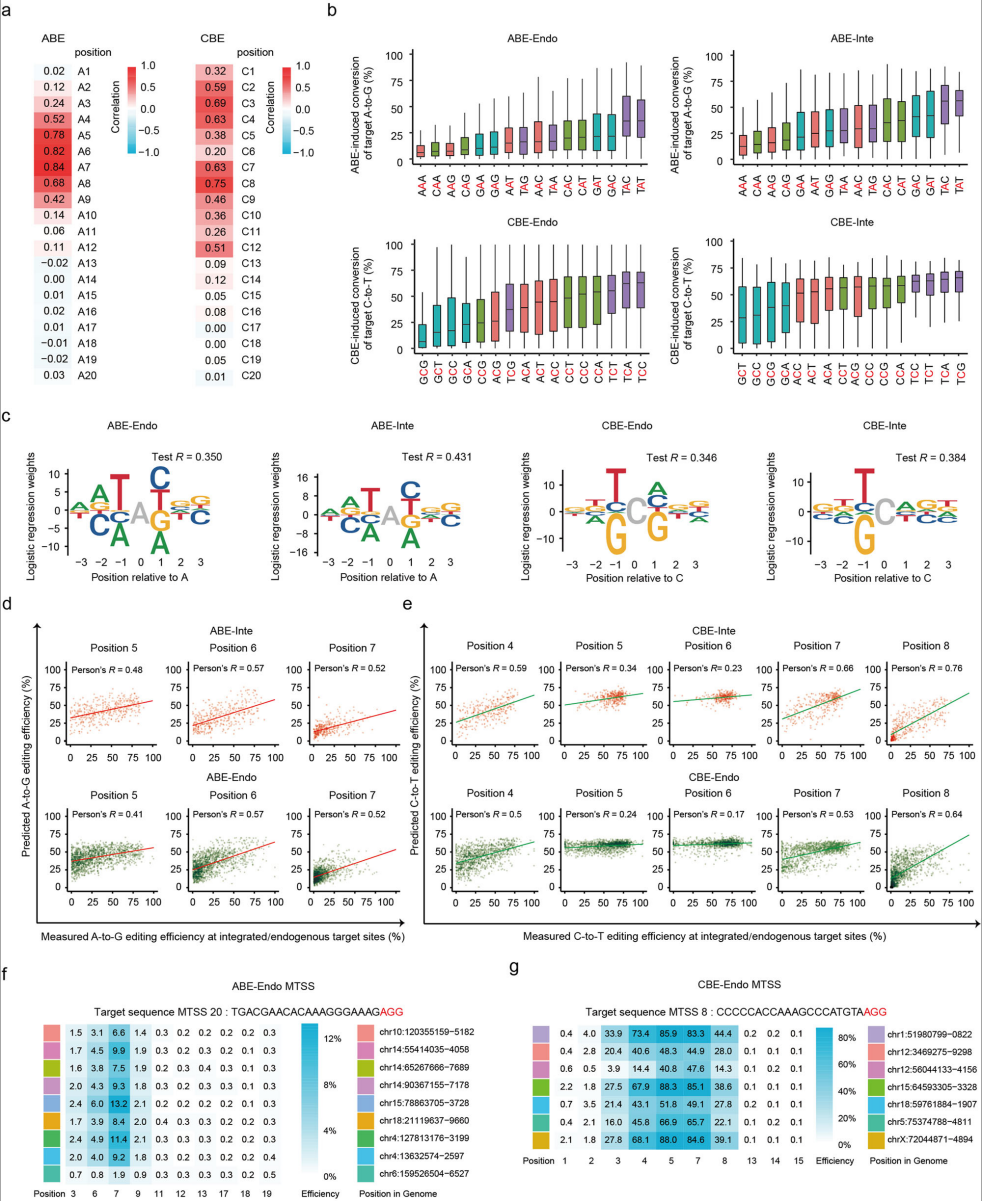
Comparison of ABE- or CBE-induced base editing efficiency between endogenous and integrated target sites. **a** Heatmap of correlation of ABE-induced A-to-G or CBE-induced C-to-T base editing efficiency between the Endo- and Inte-datasets at each protospacer positions 1–20 (PAM is at positions 21–23). *n* = 509 (position 1), 930; 1607, 809, 1343, 1250, 1106, 916, 916, 1157, 1269, 1493, 898, 1109, 1300, 1234, 936, 771, 910 and 1008 (position 20) for ABE. *n* = 576 (position 1), 1060, 563, 1025, 1181, 1040, 1195, 1343, 1082, 1079, 1065, 975, 1357, 1163, 938, 1156, 1136, 1889, 931 and 572 (position 20) for CBE. **b** Effect of the sequence context surrounding the target As or Cs (red) on the ABE- or CBE-directed base editing efficiency at endogenous and integrated target sites. *n* = 139 (AAA), 250 (AAC), 273 (AAG), 100 (AAT), 255 (CAA), 325 (CAC), 575 (CAG), 245 (CAT), 286 (GAA), 334 (GAC), 338 (GAG), 205 (GAT), 52 (TAA), 178 (TAC), 84 (TAG) and 60 (TAT) for ABE. *n* = 582 (ACA), 463 (ACC), 213 (ACG), 413 (ACT), 537 (CCA), 284 (CCC), 159 (CCG), 328 (CCT), 585 (GCA), 454 (GCC), 186 (GCG), 461 (GCT), 400 (TCA), 327 (TCC), 124 (TCG) and 268 (TCT) for CBE. **c** Sequence motifs for ABE- and CBE-directed base editing efficiency in the Endo- and Inte- datasets from logistic regression models. **d**, **e** Correlation between observed and predicted base editing efficiency from logistic models based on the ABE-Inte (**d**) or CBE-Inte (**e**) dataset. *n* = 403 (position 5), 375 and 331 (position 7) for ABE-Inte. *n* = 1342 (position 5), 1248 and 1105 (position 7) for ABE-Endo. *n* = 308 (position 4), 355, 312, 358 and 403 (position 8) for CBE-Inte. *n* = 1025 (position 4), 1181, 1040, 1194 and 1343 (position 8) for CBE-Endo. **f**, **g** ABE-directed A-to-G (MTSS 20) (**f**) or CBE-directed C-to-T (MTSS 8) (**g**) editing efficiency at representative MTSS sites. The target sequence of each MTSS site occurs 2–14 times in the genome.

Comparison of the base editing efficiency at each position between endogenous and integrated datasets demonstrated a high correlation for ABE but a low one for CBE, especially at positions 5 (*R* = 0.38) and 6 (*R* = 0.20) (Fig. 2a; Supplementary Fig. S3a, b). The C5 and C6 are two loci most likely to be edited by CBE, and the editing efficiencies were relatively high in CBE-Inte dataset, while spanned a wider range in the endogenous datasets, resulting in the relatively low correlation values between the two datasets (Fig. 1d). The higher editing efficiency at the integrated sites was possibly caused by the fact that the lentivirus preferentially integrated into genomic loci with high chromatin accessibility and the lower editing efficiency at the endogenous target sites was possibly caused by the influence of endogenous factors. To further elucidate the effect of the sequences around the target base on the BEs in the endogenous sites, the BE-directed editing efficiency was evaluated for all 16 possible sequence motifs (NAN/NCN, N = A, T, G or C) within the editing window. For ABE, the highest A-to-G conversion efficiency was observed at TAY motif (Y = C or T) while the lowest at MAR motif (M = A or C, R = A or G) (Fig. 2b; Supplementary Fig. S4a, b). These observations are consistent with those found in ABE-Inte dataset (Fig. 2b; Supplementary Fig. S4a, b) and in a previous study for ABE7.10, which used a lentiviral integrated library^9^. The A-to-G editing efficiency showed a high correlation between ABE-Endo and ABE-Inte datasets in the majority of sequence motifs except for TAT, which represents the most common motif for ABE (Supplementary Fig. S4c), suggesting that endogenous editing is different from the integrated library in a motif-dependent manner. For CBE, a C-to-T conversion efficiency was higher at TCN motif and lower when 5’ was occupied by a G in both datasets (Fig. 2b; Supplementary Fig. S4d). On the other side, the base editing efficiency at NCG motif within CBE-Endo dataset was the lowest, and much lower than (~2-folds) CBE-Inte dataset, especially for TCG and GCG motifs (Fig. 2b; Supplementary Fig. S4e, f). The C-to-T editing efficiency values at TCN motif ranged from 0% to 100% in CBE-Endo dataset, but from 20% to 80% in the integrated dataset (Fig. 2d). A low correlation of C-to-T editing efficiency was observed at TCN and NCG motifs between CBE-Endo and CBE-Inte datasets, which may also contribute to the observed low correlation between the 2 datasets at positions 5 and 6 (Supplementary Fig. S4f). In this regard, since CG-enriched motifs are typically methylated, the C-to-T base editing efficiency at endogenous sites might be affected by DNA methylation, as reported in a previous study^29^.

Logistic regression models were constructed to identify preferential target motifs for BEs using datasets with (randomly selected) 70% and 30% of the target sites for the training and validation datasets, respectively^30^. Results from this study showed that 12.3% and 12.0% of the base editing efficiency is related to the sequence motifs in ABE- and CBE-Endo datasets, respectively, as compared to 18.5% and 14.7% observed in the corresponding integrated datasets (variance = Square of Test *R*; Fig. 2c)^11^. The logistic regression model developed with ABE-Inte dataset showed similar performances in predicting A-to-G editing efficiency in both endogenous and integrated target sites (*R* = 0.41–0.57 for targeted base within the editing window) (Fig. 2d). However, the logistic regression model based on CBE-Inte dataset did not predict efficiently the C-to-T mutation within endogenous target sites, especially at positions 5 (*R* = 0.24) and 6 (*R* = 0.17) (Fig. 2e), suggesting that the editing efficiency of CBE at endogenous sites may be affected by factors other than DNA sequences.

To explore the relationship between the editing efficiency and endogenous factors, we analyzed the editing efficiencies at 22 multiple target single spacer (MTSS) sites (Supplementary Table S1), 2–14 of which were shared by the same protospacer^31^. The editing efficiency varied at different genomic loci showing identical target sequences within (up to 24-fold for ABE and 13-fold for CBE) and outside (up to 7-fold for ABE and 53-fold for CBE) the editing window for both ABE and CBE (Fig. 2f, g; Supplementary Figs. S5 and S6). These findings directly proved that the conversion efficiency of BEs was determined by endogenous factors other than target DNA sequences.

### Characterization of the effects of endogenous factors on base editing efficiency

**Fig. 3:**
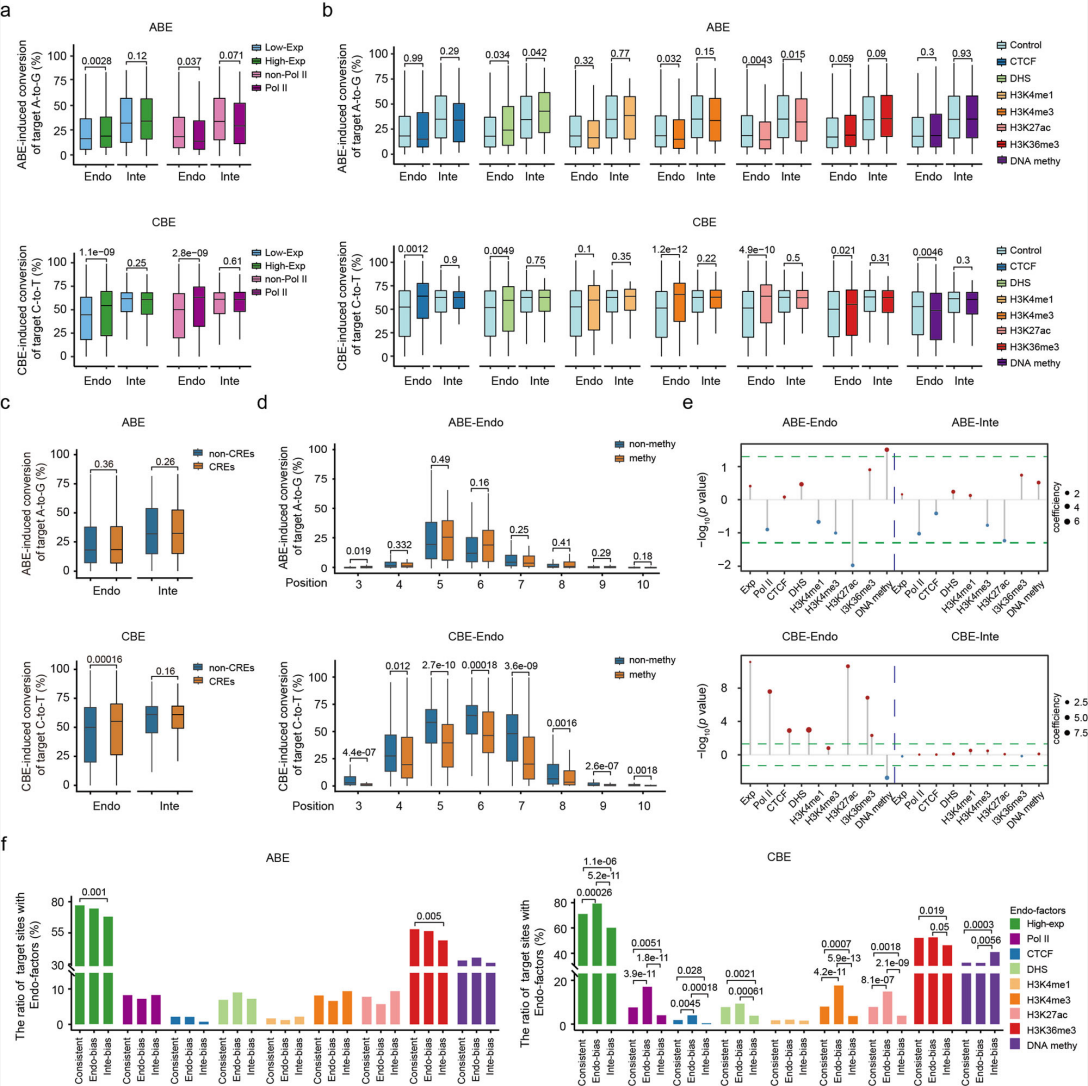
The effect of endogenous factors on ABE- or CBE-directed base editing efficiency. **a** Comparison of ABE- and CBE-directed editing efficiency at target sites with high or low gene activities within the Endo- and Inte- datasets. Exp, expression. Non, none. *n* = 700 (low-expression), 2206 (high-expression), 2670 (non-Pol II) and 236 (Pol II) for ABE. *n* = 1015 (low-expression), 2435 (high-expression), 3159 (non-Pol II) and 291 (Pol II) for CBE. **b** Comparison of ABE- and CBE-directed editing efficiency at target sites with or without the indicated epigenetic modifications in the Endo- and Inte- datasets. DHS, DNase I hypersensitive site. methy, methylation. *n* = 2848 (non-CTCF), 58 (CTCF), 2698 (non-DHS), 208 (DHS), 2858 (non-H3K4me1), 48 (H3K4me1), 2671 (non-H3K4me3), 235 (H3K4me1), 2683 (non-H3K27ac), 223 (H3K27ac), 1253 (non-H3K36me3), 1653 (H3K36me3), 1949 (non-methy) and 957 (methy) for ABE; *n* = 3384 (non-CTCF), 66 (CTCF), 3195 (non-DHS), 255 (DHS), 3391 (non-H3K4me1), 59 (H3K4me1), 3148 (non-H3K4me3), 302 (H3K4me1), 3165 (non-H3K27ac), 285 (H3K27ac), 1682 (non-H3K36me3), 1768 (H3K36me3), 2286 (non-methy) and 1164 (methy) for CBE. **c** Comparison of ABE- and CBE-directed editing efficiency at target sites with or without CRE-binding sites in the Endo- and Inte- datasets. *n* = 680 (CREs) and 2224 (non-CREs) for ABE. *n* = 826 (CREs) and 2618 (non-CREs) for CBE. **d** Comparison of ABE- and CBE-directed editing efficiency at positions 3–10 of each protospacer for endogenous target sites with or without DNA methylation. *n* = 48 (position 3), 34, 39, 56, 52, 29, 50 and 34 (position 10) for ABE methylated. *n* = 1779 (position 3), 907, 1487, 1372, 1205, 1045, 1004 and 1308 (position 10) for ABE non-methylated. *n* = 66 (position 3), 87, 103, 76, 102, 113, 63 and 110 (position 10) for CBE methylated. *n* = 580 (position 3), 1088, 1260, 1088, 1269, 1417, 1169 and 1119 (position 10) for CBE non-methylated. **e** Coefficients and *P* values of each endogenous factor in the linear regression between CBE or ABE base editing efficiency with the number of Cs or As as confounding factors. **f** Ratio of target sites with endogenous factors among “Consistent”, “Endo-bias”, and “Inte-bias” groups for ABE and CBE. Endo-factors, endogenous factors.

It was reported that epigenetic factors may influence the cleavage activity of CRISPR-Cas9 systems in eukaryotic cells^15–19^. To explore the association between endogenous factors and editing efficiency of BEs, endogenous factors for each site were quantified based on their genomic loci (Supplementary Fig. S7 and Table S2)^20–23^, and it was investigated to which extent the editing efficiency of BEs was affected by each of the endogenous factors. We observed that the target sites with a high transcriptional activity showed significantly higher A-to-G editing efficiency than sites with low transcriptional activity within the ABE-Endo dataset (Fig. 3a). Finally, to rule out the influence of different target sequences between high- and low-transcriptional groups, we also separated integrated target sites into high- and low-transcriptional groups according to transcriptional activity of their corresponding endogenous target sites, and no significant differences were observed between the 2 groups (Fig. 3a). These results suggested that the differences in the editing efficiency are caused by different transcriptional activities of the 2 groups within the Endo-dataset. Similarly, the C-to-T editing efficiency was also found to be significantly associated with transcriptional activity of targeted genes in CBE-Endo dataset (Fig. 3a), in line with the influence of the transcriptional activity on the cleavage efficacy of CRISPR-Cas9 systems^32^. Besides, it is well known that RNA polymerase II (Pol II) plays an essential role in regulating gene expression^33^, and endogenous target sites with Pol II binding exhibited significantly higher base editing efficiency for CBE (Fig. 3a; Supplementary Fig. S8). Although endogenous target sites with Pol II binding demonstrated a lower A-to-G base editing efficiency than those without, the two groups showed no significant differences in the subsampling analysis, which was performed to remove the bias from uneven numbers of target sites between groups (see Methods; Supplementary Fig. S8).

As for the epigenetic factors, CCCTC-binding factor (CTCF) binding to promoters could induce a long-distance enhancer-dependent transcription in several cell types^34^; target sites with CTCF binding showed higher C-to-T editing efficiency in CBE-Endo dataset (Fig. 3b; Supplementary Fig. S8). In addition, target sites with high chromatin accessibility (targets at DNase I hypersensitive regions, DHS), showed a significantly higher C-to-T editing efficiency in CBE-Endo dataset (Fig. 3b; Supplementary Fig. S8), in line with previous reports stating that chromatin accessibility may influence the binding and cleavage activity of Cas9^16,35^. We also demonstrated that target sites with histone modifications, including H3K4me3, H3K27ac, and H3K36me3, had significantly higher C-to-T editing efficiency in CBE-Endo dataset (Fig. 3b; Supplementary Fig. S8), while H3K4me1 modifications showed no significant correlation with C-to-T editing efficiency at the endogenous sites (Fig. 3b; Supplementary Fig. S8). By contrast, most of epigenetic factors showed no significant association with the editing efficiency of ABE at endogenous target sites except for chromatin accessibility and H3K27ac variables (Fig. 3b; Supplementary Fig. S8). By integrating DNA accessibility and chromatin modification data into CREs according to the categorization of the Encyclopedia of DNA Elements (ENCODE)^36^, we found that endogenous target sites with CREs exhibited a significantly higher C-to-T editing efficiency than those without, while no significant differences in A-to-G editing efficiency was described between target sites regardless of the presence of CREs (Fig. 3c).

Besides histone modifications, DNA 5-mC at CG sequence motifs (CpG islands) represents one of the most studied epigenetic factors associated with gene silencing^37^. Exploring the influence of DNA methylation on the base editing efficiency, we found that the editing efficiency was significantly lower for CBE but not for ABE at the target sites with DNA methylation (Fig. 3b). Further examination revealed that targeted Cs with DNA methylation showed a significantly lower C-to-T editing efficiency than those without, suggesting that the influence of DNA methylation may be restricted to methylated Cs (Fig. 3d). In addition, the editing efficiencies of C5 and C6 loci showed most drastic decrease under DNA methylation, possibly explaining the low correlation in base editing efficiencies with CBE-Inte dataset (Fig. 2a; Supplementary Fig. S3b). Moreover, the linear regression for each endogenous factor with the number of targeted Cs as confounding factor also showed that the editing efficiency of CBE had a positive correlation with transcriptional activity, Pol II and CTCF binding, DHS, H3K4me3, H3K27ac, and H3K36me3 but a negative association with DNA methylation (Fig. 3e). Unexpectedly, DNA methylation and H3K27ac showed positive and negative contribution to the A-to-G editing efficiency, respectively, following the regression of targeted As in the ABE-Endo dataset, contrarily to that observed for the CBE-Endo dataset (Fig. 3e). Further examination showed that the targeted As with DNA methylation had significantly higher proportion of ABE-preferential motifs, suggesting that the DNA-methylation associated with a high efficiency actually resulted from the underlying preferred motifs of ABE (Supplementary Fig. S9a). Since it is well known that modified histones might involve larger regions with one or multiple nucleosomes, further analysis involving the broader regions flanking the target region revealed that H3K27ac was associated with a lower editing efficiency for A-to-G editing within endogenous sites (Supplementary Fig. S9b).

To further investigate the role of endogenous factors in leading the differences of editing efficiency between the endogenous and integrated datasets, the target sites were divided into three groups, including “Consistent”, “Endo-bias” and “Inte-bias” groups, based on the consistency of the editing efficiency between the 2 datasets (Supplementary Fig. S10a). The “Consistent” group included target sites with similar efficiency between the two datasets, like the “Endo-bias” and “Inte-bias” groups (Supplementary Fig. S10b). As expected, the proportion of target sites with endogenous factors, including high transcriptional activity, Pol II and CTCF binding, DHS, and H3K27ac, H3K36me3 and H3K4me3 histone modifications, was highest in CBE “Endo-bias” group and lowest in CBE “Inte-bias” group (Fig. 3f). On the contrary, the proportion of target sites with DNA methylation was highest in CBE “Inte-bias” group, compared with other 2 groups. Comparing the consistency between the 3 groups of ABE datasets, no big differences were observed and only the proportion of target sites with high transcriptional activity and H3K36me3 modification in ABE “Inte-bias” group was lower than that in the Consistent group (Fig. 3f). These results together directly demonstrated that endogenous factors, including transcriptional activity, chromatin accessibility, DNA and histone modifications, may influence the activity of BEs at endogenous genomic sites, resulting in a different conversion efficiency of ABE and CBE.

### Comparison of editing outcomes of BEs between endogenous and integrated target sites

**Fig. 4:**
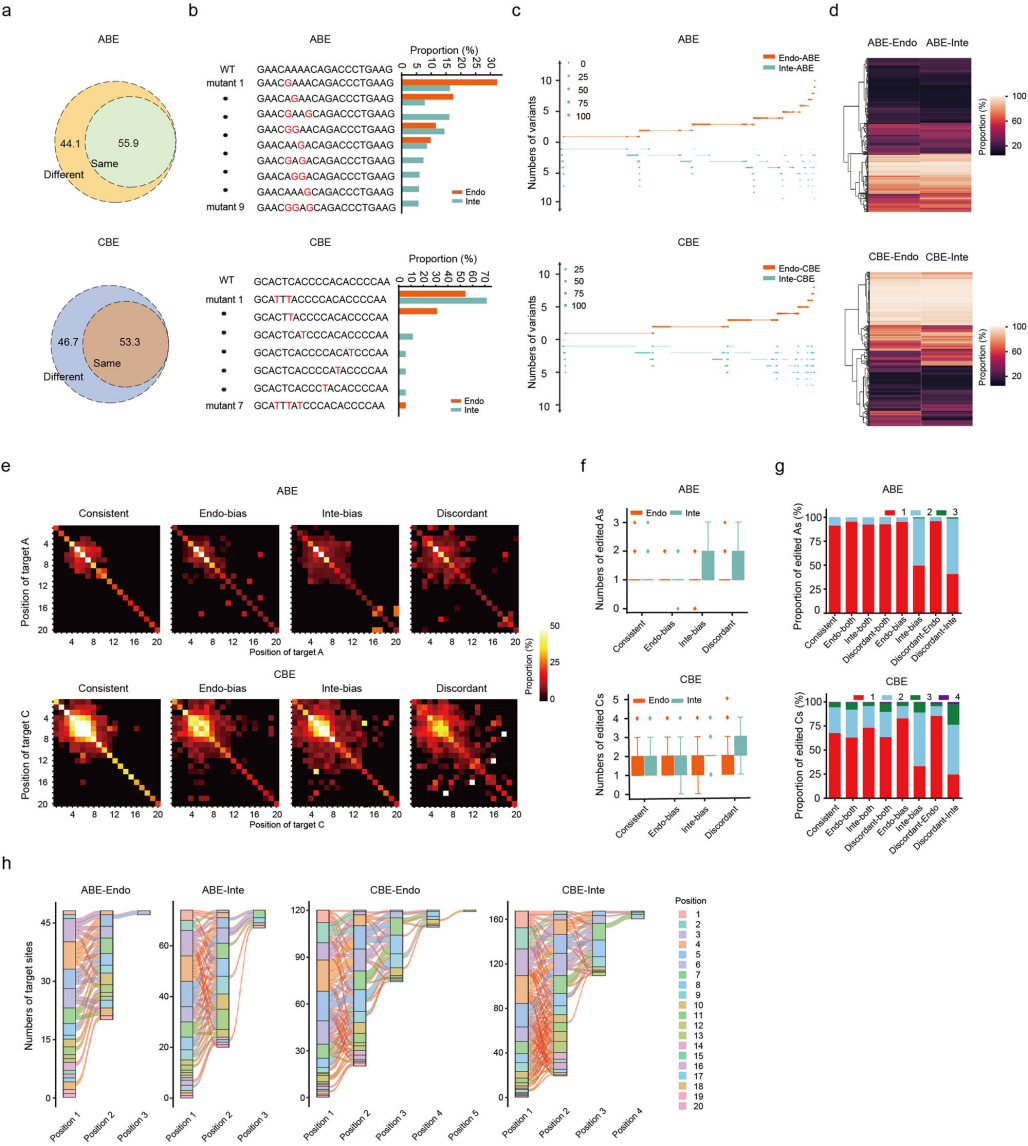
Comparison of editing outcomes of ABE and CBE between endogenous and integrated target sites. **a** Comparison of the editing outcome alleles between the Endo- and Inte- datasets for ABE and CBE. **b** Representative editing outcomes of ABE or CBEs at endogenous and integrated target sites with the same sequence. **c** Comparison of the editing outcome products between the Endo- and Inte-datasets for ABE and CBE. The dot size represents percentage of unique editing outcome products in Endo- or Inte- dataset. The target sites were thus divided into 4 groups: “Consistent”, target sites with consistent editing outcomes between the Endo- and Inte- datasets; “Endo-bias” and “Inte-bias” groups, target sites with specific alleles in the Endo- or Inte- datasets, respectively; “Discordant” group, target sites with specific alleles in both Endo- and Inte-datasets. **d** Comparison of the proportions of same editing outcome products in the consistent group of ABE and CBE. **e** Co-occurrence proportions of each targeted As or Cs. **f** Comparison of the number of edited As (ABE) or Cs (CBE) within each target site among the “Consistent”, “Endo-bias”, “Inte-bias”, and “Discordant” groups of ABE or CBE. *n* = 2005 (Consistent), 824 (Endo-bias), 511 (Inte-bias) and 245 (Discordant) for ABE. *n* = 1930 (Consistent), 877 (Endo-bias), 552 (Inte-bias) and 263 (Discordant) for ABE. **g** Normalized proportion of edited As (ABE) or Cs (CBE) within each target site among the “Consistent”, “Endo-bias”, “Inte-bias”, and “Discordant” groups of ABE or CBE. **h** Preference of position flow for targeted As or Cs in the protospacer of target sites for Endo-specific and Inte-specific editing outcomes.

As for the applications of base editing in reversing the pathogenic point mutations and generating animal models, only precise editing at specific targeted C or A is desired and editing at unintended bases may lead to unwanted phenotypes^38,39^. Thus, it is important to examine the editing outcomes to avoid unwanted edits at the target sites. When comparing the base editing outcomes for each target sequence between the endogenous and integrated datasets for BEs, we found that nearly 50% of target sites had different editing outcome products (Fig. 4a–c). We then classified the target sites into “Consistent”, “Endo-bias”, “Inte-bias” and “Discordant” groups, representing those with editing outcome products consistent between the two datasets, unique to Endo- or Inte- dataset, and discordant between the two datasets, respectively (Fig. 4c). Although the forms of editing outcome products were same in the “Consistent” group, proportions of the same editing product still varied a lot between the endogenous and integrated datasets (Fig. 4d), indicating the discordance in base editing outcome products of the two datasets.

Interestingly, the co-occurrent editing proportions for targeted As or Cs at different positions were higher in the “Discordant” than the “Consistent” group (Fig. 4e). Further analysis revealed that when the target sequences contained more than one A or C, BEs tended to edit more bases in the Inte-datasets instead of in the Endo-datasets (Fig. 4f, g). Position flow analysis for targeted As or Cs between Endo-specific and Inte-specific editing outcomes further showed that BEs tended to edit more As or Cs at the same time in the Inte-datasets than in the Endo-datasets, resulting in more diverse forms of editing outcome products (Fig. 4h). Together, by pairwise-comparison of the base editing outcome products between endogenous and integrated target sites with the same sequences, we found that the forms and proportions of base editing outcomes varied a lot between the two datasets, especially when the target sequences contained more than one A or C. These results directly suggested the necessity of development of deep learning models using endogenous dataset for accurate base editing outcome prediction at endogenous target sites.

### Development of deep learning models incorporating endogenous factors to predict the base editing outcomes

**Fig. 5:**
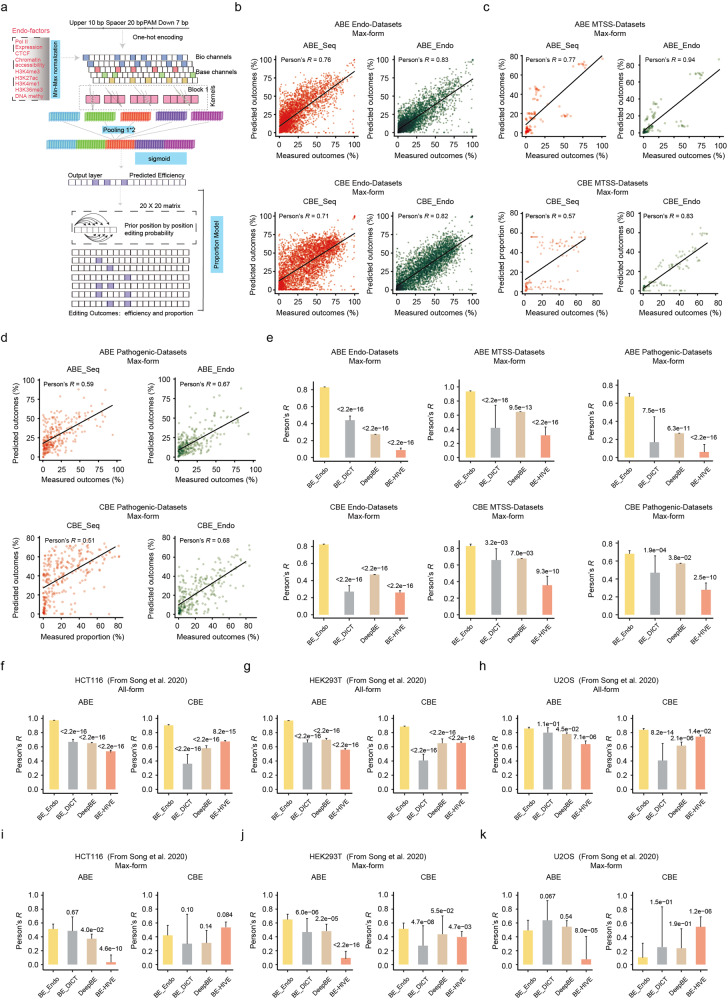
Development and evaluation of deep learning models for prediction of editing outcomes of BEs. **a** Schematic representation of deep learning methods development by integrating sequences features with endogenous factors in order to predict base editing efficiency and allele proportions. **b**–**d** Performance evaluation of BE_Seq and BE_Endo models for predicting desired form of edits using testing endogenous (**b**), independent MTSS (**c**) and Pathogenic (**d**) datasets. **e** Performance evaluation of different models for ABE and CBE on prediction of the Max-form edited outcomes using all 3 datasets from this study. *n* = 4131 (Endo) for ABE and 4116 (Endo) for CBE. *n* = 120 (MTSS) for ABE and 118 (MTSS) for CBE. *n* = 292 (pathogenic) for ABE and 274 (pathogenic) for CBE. **f–k** Performance evaluation of different models for ABE and CBE on prediction of the All-form (**f–h**) and Max-form (**j,** **k**) of edited outcomes at endogenous target sites in different cell lines using the published datasets from Song et al. 2020. *n* = 276 (HCT116), 578 (HEK293T) and 148 (U2OS) for ABE (All-form of edited outcomes). *n* = 278 (HCT116), 704 (HEK293T) and 186 (U2OS) for CBE (All-form of edited outcomes). *n* = 41 (HCT116), 94 (HEK293T) and 27 (U2OS) for ABE (Max-form of edited outcomes). *n* = 35 (HCT116), 100 (HEK293T) and 28 (U2OS) for CBE (Max-form of edited outcomes). *P* values above each group were calculated in comparison with the “BE_Endo” models.

Taking the effects of endogenous factors on the editing efficiency of BEs into account, a deep learning model which incorporated these features was developed to optimize the sgRNA selection. In details, a hybrid deep neural network was designed to predict the per-base A-to-G or C-to-T editing efficiency using both a protospacer target sequence and endogenous factor at each target site (Fig. 5a). The model takes a 40-bp target sequence (10 bp upstream + 20 bp protospacer + 3 bp PAM + 7 bp downstream) as input, while the endogenous features of each target site were also added to the model (see Materials and methods; Fig. 5a). The 80% of ABE or CBE datasets were used for model training, while for performance evaluation the 20% of each model was used. Pearson correlation coefficient (*R*) was used to assess the performance of each model. The models based on sequence features trained using Inte-datasets were named as ABE_Seq and CBE_Seq efficiency models and these models showed a significant decreased prediction performance in the Endo-datasets with shared target sequences (Supplementary Fig. S11a, b), suggesting an influence of factors other than sequence contents in determining the base editing efficiencies at endogenous target sites. So, we developed deep learning models of ABE and CBE, incorporating one or all of endogenous factors (Supplementary Fig. S11c, d). As H3K27ac modification plays a role in influencing the A-to-G editing efficiency, the model integrating H3K27ac modification, named ABE_Endo efficiency, was selected for further analysis (Fig. 3; Supplementary Figs. S9a, b and S11c). Considering the influence of all endogenous factors on the C-to-T editing efficiency, the model integrating all factors was designated as CBE_Endo efficiency and used for further testing analysis (Fig. 3; Supplementary Fig. S11d).

Next, we evaluated the performance of BE_Endo efficiency models in predicting base editing efficiency in the independent MTSS dataset, and found that the BE_Endo efficiency models showed a significantly higher accuracy in predicting editing efficiency of target bases within editing window than those obtained with BE_Seq models (Supplementary Fig. S11e, f). For facilitating the application of BEs in clinical research, we also applied the models to predict the efficiencies of base editing outcomes for the pathogenic target sites of human disease-relevant point mutations reported in ClinVar4^39^. Specifically, we searched for pathogenic and likely pathogenic point mutations that could be generated using an editable window (positions 5–7 for ABE and 4–8 for CBE) with an NGG PAM. This exploration identified 299 and 296 target sites (only one A or C located in the editing windows) that could theoretically be mutated to pathogenic point mutations by ABE and CBE, respectively (Supplementary Table S1). Compared with the BE_Seq efficiency models, BE_Endo efficiency models showed a higher prediction accuracy at the pathogenic dataset (Supplementary Fig. S11e, f). In addition, we also generated a new endogenous dataset with 201 target sites located in the intergenic regions of the genome and found that CBE_Endo efficiency model outperformed CBE_Seq efficiency model (Supplementary Fig. S11f). Moreover, CBE_Endo efficiency model also showed improved predicting accuracy in another independent test dataset from a previous study (Supplementary Fig. S11f)^9^. In order to determine the influence of each factor in prediction accuracy, we compared the performance of BEs_Seq and BEs_Endo efficiency models at target sites with specific factor using different datasets. For the Endo-datasets, BE_Endo efficiency models outperformed BE_Seq efficiency models at target sites with each endogenous factor examined, especially for CBE (5.76%–16.83%; Supplementary Fig. S11g). For the other 4 independent testing datasets, CBE_Endo efficiency model showed dramatically improved prediction accuracy than CBE_Seq efficiency model at target sites with expression, DNA methylation, H3K36me3 or Pol II modification (13.00%–56.11% for MTSS, 13.27%–28.53% for Pathogenic, 14.47% for Intergenic and 11.38%–32.42% for HEK293T^9^; Supplementary Fig. S11h–k). ABE_Endo efficiency model showed only slight influence on prediction accuracy at target sites with endogenous factors in the examined datasets (Supplementary Fig. S11h–k). These results indicated that endogenous factors have significant influence on CBE editing efficiency in different endogenous datasets, with little impact on the ABE editing. In the 4 independent testing datasets, the prediction accuracy of CBE_Endo efficiency model increased with the proportions of target sites with endogenous factors (Supplementary Fig. S11l).

Moreover, compared with logistic regression models (Person’s *R* = 0.41–0.57 for ABE and 0.17–0.64 for CBE; Fig. 2d, e), BE_Seq efficiency models, based on sequence features, performed better in predicting the base editing efficiency at endogenous target sites (Person’s *R* = 0.64–0.75 for ABE and 0.21–0.71 for CBE; Supplementary Fig. S12a, b). The deep learning models integrating endogenous factors, BEs_Endo efficiency models, further improved prediction accuracy, especially for CBE at positions 5 (1.80-fold) and 6 (2.71-fold) (Person’s *R* = 0.64–0.78 for ABE and 0.57–0.82 for CBE; Supplementary Fig. S12a, b). CBE_Endo efficiency model also outperformed CBE_Seq efficiency model in predicting the C-to-T efficiency at TCN or NCG motifs, and low correlation was observed between Endo- and Inte-datasets (10%–62%; Supplementary Fig. S12c).

To further predict the proportions of editing outcome products of each endogenous target site, we further applied a Bayesian network to infer the dependency between each of the two targeted nucleotides. Firstly, we calculated the prior correlations between each pair of the target base and found that the editing outcomes of ABE or CBE in the endogenous datasets varied a lot from in the integrated datasets (Supplementary Fig. S13a, b). Given that the majority of the information between distant editing positions was accounted for by joint probability with the adjacent editing position, we applied a simplified Bayesian network model to calculate the joint probabilities of any possible editing outcomes for each target site (see Materials and methods; Supplementary Fig. S13c). Based on the BE_Endo efficiency models, we then developed the BE_Endo proportion models to predict the proportions of different editing outcomes of ABE and CBE at the endogenous target sites (Fig. 5a). To facilitate the utility of these models, we combined ABE_Endo efficiency with proportion models, generating ABE_Endo. CBE_Endo was generated in a similar way, by combining CBE_Endo efficiency with proportion models (freely accessible web tools are available at http://www.sunlab.fun:3838/BE_Endo). Next, evaluating the prediction performance of BE_Endo models in the testing endogenous, and independent MTSS and pathogenic datasets, we found that BE_Endo outperformed BE_Seq in all 3 datasets for validation in predicting all the outcomes (termed as “All-form”) (Supplementary Fig. S14a–c). Base editing usually generates different forms of editing outcomes with A-to-G for ABE or C-to-T for CBE (termed as “Edited-form”), while only those with precise editing at specifically targeted As or Cs were desired. Notably, the prediction accuracy with the highest base editing frequency among the Edited-forms (termed as “Max-form”) obtained using BE_Endo showed a significantly higher correlation within the Endo, independent MTSS and pathogenic datasets, especially for CBE, when compared with that obtained with BE_Seq (Fig. 5b–d). Recently, several computational methods have been developed for predicting the editing outcomes of BEs, such as BE-HIVE, DeepBE and BE-DICT^9,11,13^. Compared with these 3 models, BE_Endo showed similar performance in predicting the editing outcomes using the integrated testing datasets from corresponding research (Supplementary Fig. S14d, e). BE_Endo also outperformed these 3 models in predicting Max-form and All-form of outcomes using Endogenous, MTSS and Pathogenic datasets (Fig. 5e; Supplementary Fig. S14f–h). To demonstrate the generality of BE-Endo, we next compared the prediction accuracy of different models using 3 endogenous datasets from the corresponding studies^4^ and found that BE_Endo outperformed the other 3 models in predicting All-form of outcomes (Fig. 5f–h). For predicting Max-form of edited outcomes, BE_Endo also showed better or comparable performance in HCT116 and HEK293T cells (Fig. 5i–k). These results directly demonstrated the more accurate prediction of base editing outcomes of BE_Endo models, which integrated endogenous factors.

## Discussion

In this study, the similarities and differences of base editing outcomes of ABE and CBE at thousands of target sites were compared with the same sequences between endogenous and integrated datasets. The effects of endogenous factors on BEs were investigated and deep learning models were developed to achieve more accurate prediction for endogenous base editing.

Recent studies developed computational models to predict editing outcomes through high-throughput datasets^9,11–13^. The models are beneficial for the sgRNA optimization of genome editing and should be used for future applications^9,11–13^. However, the editing outcomes of high-throughput datasets were measured in a synthetic environment, which led to a low correlation with endogenous genomic editing. In addition, the labor- and time-consuming process to measure the editing outcomes of each sgRNA resulted in very small endogenous datasets before^9,10^. In this study, for the first time we developed genome-wide endogenous datasets including over 5000 target sites, which included also genome-integrated datasets containing the same target sequences as pairwise comparisons. The comparative analyses revealed that the editing outcomes, including product purity, indels, editing efficiency and proportions, varied a lot between endogenous and integrated target sites. The higher editing efficiency at the integrated sites was possibly caused by the fact that the lentivirus preferentially integrated into genomic loci with high chromatin accessibility^40^, which are areas more accessible to BEs. Therefore, the assays using the genome-integrated synthetic datasets likely overestimated the editing efficiency of BEs at endogenous target sites.

The genome-wide datasets also allowed us to systematically evaluate the effects of the endogenous factors on the BEs editing. We found that transcriptional activity, Pol II and CTCF binding, chromatin accessibility, DNA and histone modifications were associated with the editing efficiency of ABE and CBE. Generally, target sites with high transcriptional activities showed higher base editing efficiency for both ABE and CBE, while target sites with chromatin accessibility were associated with higher C-to-T but not with A-to-G editing efficiency, indicating that the CBE binding to the target sequence was more affected by the position of the nucleosome, consistently with previous reports about CRISPR/Cas9^16,18,35^. In addition, since the nucleosome is composed of DNA wound around histone proteins^41^, specific histone modifications exert different effects on the editing outcomes of ABE and CBE. Specifically, histone modifications H3K4me3, H3K27ac, and H3K36me3 promoted the C-to-T instead of the A-to-G editing efficiency. Indeed, the influence of the histone modifications on base editing may be related to their role in regulating gene expression and chromatin accessibility^42^. The different influence of endogenous factors on editing efficiency of ABE and CBE, may be due to the fact that rAPOBEC1 was a pre-existed cytosine deaminase of ssDNA in mammal cells and TadA evolved from a tRNA^Arg^ adenine deaminase of *E.coli*^2^. TadA of ABE can catalyze adenine deamination in both ssDNA and dsDNA, but rAPOBEC1 of CBE catalyzes cytosine deamination in ssDNA^43^. The preference of different catalytic substrates between ABE and CBE may lead to higher editing efficiency of CBE at target sites with high chromatin accessibility and low DNA methylation. During the progression of deamination, TadA catalyzes A to inosine (I) conversion and rAPOBEC1 catalyzes C to uracil (U) conversion, which are read as G and T by polymerases, respectively^44^. Following deamination progression, uracil excision is much more efficient than inosine excision, and a uracil glycosylase inhibitor protein (UGI) is added to the C-terminal of CBE to increase the half-life of uracil at the target locus. An additional UGI of CBE may influence recruiting proteins of mismatch repair to the target sites, resulting in distinct performance between CBE and ABE at endogenous target sites.

Incorporating sequence features and endogenous factors of great importance, we developed BE_Endo models for predicting the efficiency and proportion of editing outcomes of BEs. Compared with BE_Seq models, BE_Endo showed dramatically improved prediction performance, especially for CBE. These observations also proved that activity of CBE was more likely to be affected by endogenous factors beyond sequence, when compared with ABE. Although our study clearly proved that specific endogenous factors may influence the base editing outcomes of endogenous target sites for both ABE and CBE, limited information is available on the epigenetic factors. Therefore, further studies aiming at investigating the genome context will better elucidate the genomic features that influence the editing outcomes of BEs. In addition, for the proportion models of CBE and ABE, we used a simplified Bayesian network to calculate the joint probabilities for all possible editing events given the conditional independence between distant editing positions for base editing. The relationship between distant editing positions could be directly taken into consideration for proportional models in future studies.

## Materials and methods

### Plasmids

The coding sequences of ABE and CBE were cloned from pCMV-ABEmax (Addgene plasmid #112095) and pCMV-YE1-BE3-FNLS (Addgene Plasmid #154005), respectively. Site-directed mutagenesis of ABEmax was generated using site specific primers, and then the ABEmax^F148A^-P2A-GFP or YE1-BE3-FNLS-P2A-GFP expression cassette was cloned into plasmids containing PiggyBac transposon using NEBuilder HiFi DNA Assembly Master Mix (NEB) according to standard protocols. Each 20-bp sgRNA spacer (Supplementary Table S1) was cloned into pLenti-guide-puro (Addgene Plasmid #52963) using enzyme *BsmB*I digestion.

### Cell lines

Human embryonic kidney (HEK) 293 T cells (ATCC #CRL-3216) were cultured in Dulbecco’s modified Eagle medium (DMEM, Gibco) supplemented with 10% fetal bovine serum (FBS, BI) and 1% penicillin/streptomycin (Gibco) at 37 °C in 5% CO_2_ incubators. For PiggyBac transposon-mediated base editor integration, HEK293T cells were transfected with PiggyBac transposase plasmid and transposon plasmid harboring ABEmax^F148A^ or YE1-BE3-FNLS expression cassette using Lipofectamine 3000 (ThermoFisher Scientific) according to standard protocols. The transfected cells were washed with PBS and digested with 0.25% trypsin (Gibco) 48 h after transfection. Then cells were filtered with a 40-μm cell strainer and GFP-positive cells were enriched by flow cytometer for several rounds. The gating strategy for the separation of GFP^+^ and GFP^−^ cells was supplied in Supplementary Fig. S1b.

### Measurement of editing outcomes of ABE and CBE at endogenous target sites

For evaluation of ABE- and CBE-directed editing outcomes at endogenous target sites, HEK293T cells stably expressing ABEmax^F148A^ or YE1-BE3-FNLS were seeded into 24-well dishes and transfected with 1 μg plasmid expressing guide RNA per well using polyethyleneimine (PEI, Polyscience) according to the manufacturer’s protocols. Then 24 h after transfection, untransfected cells were removed by adding 2 μg/mL puromycin (Invitrogen) to the medium, and successfully transfected cells were collected for genomic DNA extraction. The target sites of interest were amplified by 2 rounds of nested PCR using site-specific primers (Supplementary Table S1). Each round was performed at 95 °C for 3 min, 30 cycles at 95 °C for 30 s, 59 °C for 30 s, 72 °C 60 s, and a final extension at 72 °C for 5 min. The amplicons were purified using universal DNA purification kit (OMGEA) according to the manufacturer’s instructions. The PCR products were then ligated to adapters and sequencing was performed on Illumina HiSeq X Ten platform.

### Plasmid library preparation

A pool of 11,868 oligonucleotides was array synthesized (Genewiz). Briefly, each oligonucleotide contains a 20 nt guide sequence, an enzyme *BsmB*I cutting site, a 10 nt consistent sequence, an enzyme *BsmB*I cutting site, and the corresponding target sequence. The plasmid library containing guide RNA and paired target sequence were cloned into guide RNA expression plasmid by a two-step process as previously reported^18^.

Step I: Generation of plasmid library containing sgRNA spacers and their corresponding target sequences. The restricted enzyme cutting site of *EcoR*I was inserted into the backbone plasmid (Addgene Plasmid #52963) with sgRNA scaffold using NEBuilder HiFi DNA Assembly Master Mix (NEB). Then, the backbone plasmid was digested with enzymes *BsmB*I and *EcoR*I, and gel-purified using gel extraction kit. The oligonucleotide library of paired sgRNAs and target sequences was amplified using KOD polymerase (KOD). The purified amplicons and digested backbone plasmid were incubated for 1 h at 50 °C using NEBuilder HiFi DNA Assembly Master Mix. The products were then transformed into electrocompetent cells using micropulser electroporator (Bid-Rad). The library coverage was more than 500× of the number of total oligonucleotides.

Step II: Insertion of sgRNA scaffold into the plasmid library. The plasmid library from Step I was digested with enzyme *BsmB*I and gel-purified using gel extraction kit. The sgRNA scaffold sequences were cloned (a gift from Dr. Leopold Parts in Wellcome Sanger Institute) and digested with enzyme *BsmB*I. The digested plasmid library and insert fragment were ligated using T4 ligase (ThermoFisher Scientific) overnight at 16 °C. The products were transformed into electrocompetent cells using micropulser electroporator. The library coverage was more than 500× of the number of total oligonucleotides. The colonies were collected using Plasmid Maxiprep kit (QIAGEN).

### Lentivirus production

Paired sgRNA lentiviral plasmid library was produced as previously described^30^. For lentivirus production, HEK293T cells were transfected with 30 μg paired sgRNA lentiviral vector, 22.5 μg psPAX2 and 15 μg pMD2.G in a 15 cm dish using polyethyleneimine. The supernatant containing lentiviral particles was collected 48 h and 72 h after transfection, centrifuged at 4500 rpm for 15 min and filtered through a Millex-HV 0.22-μm low protein-binding membrane. The filtered supernatant was centrifuged at 27,000 rpm for 2 h, dissolved in PBS and stored at –80 °C.

### Lentiviral plasmid library transduction

The day before transduction, 3 × 10^7^ HEK293T cells stably expressing ABE or CBE were seeded into 15 cm dishes overnight. The cells were infected with paired sgRNA lentiviral plasmid library at MOI = 0.3 with 10 μg/mL polybrene. Then 24 h after infection, the untransduced cells were removed by adding 2 μg/mL puromycin into the medium, and the successfully transduced cells were collected after puromycin treatment for another 48 h. Genomic DNA was next extracted from the puromycin -resistant cells using TIANamp Genomic DNA Kit (QIAGEN) according to the manufacturer’s protocols.

### Measurement of gene editing outcomes at integrated target sites

The integrated target sequences were amplified for 2 rounds of PCR using Premix Ex Taq (Takara). A total of 240 μg genomic DNA (10 μg genomic DNA per 10^6^ cells) was used as template for the first round of PCR. The coverage of the paired sgRNA lentiviral plasmid library was 2000× for each sample. The 240 μg genomic DNA was separated into 96 50-μL reactions with 2× Premix Ex Taq, outer forward and reverse primers (Supplementary Table S1). The first round of PCR was performed at 95 °C for 3 min, 20 cycles at 95 °C for 30 s, 60 °C for 30 s, 72 °C 30 s, and a final extension at 72 °C for 5 min. The products of the first round of PCR were mixed and used as template for the second round of PCR. The 100 μL products was separated into 96 50-μL reactions with 2× Premix Ex Taq, inner forward and reverse primers (Supplementary Table S1). The second round of PCR was performed at 95 °C for 3 min, 32 cycles at 95 °C for 30 s, 60 °C for 30 s, 72 °C 30 s, and a final extension at 72 °C for 5 min. The products from the second round of PCR were purified with gel extraction kit. The purified PCR products were then ligated to adapters and sequencing was performed on the Illumina HiSeq X Ten platform.

### Deep sequencing data alignment and preprocessing

High-throughput sequencing datasets were processed using CRISPResso2^45^; the amplicon for each target site was used as reference sequence (170 bp, including 75 bp upstream of protospacer, 20 bp protospacer and 75 bp downstream of protospacer). Redundant barcodes were trimmed by cutadapt (v1.18), and clean reads were aligned to the reference using a global alignment algorithm with default parameters. The allele frequency table for each target site from CRISPResso2 was used for calculating base editing efficiency and outcomes in the following analysis.

Specifically, the overall base editing efficiency for each target site was defined as:

$$\frac{{total}\,{reads}\,{included}\,{intended}\,{base}\,{transition}\,{in}\,{the}\,{window}}{{Total}\,{reads}}* 100 \%$$

The base editing efficiency at each targeted C or A was calculated as:

$$\frac{{total}\,{reads}\,{included}\,{intended}\,{base}\,{transition}\,{at}\,{each}\,{position}}{{Total}\,{reads}}* 100 \%$$

Additionally, modified editing efficiency for each target site was considered as:

$$\frac{{Edited}\,{reads}\,{with}\,{base}\,{mutation}\,{or}\,{indels}}{{Total}\,{reads}}* 100 \%$$

Total reads were the sum of all reads aligned to the reference. To improve the accuracy of base editing, we excluded some sgRNAs with coverage less than 100, and the average efficiency among biological replicates for each target site was used for the following analysis.

### Base editing purity

Unintended base editing events were previously reported in base editors^11^. To account for the efficiency of each base transition, all the editing outcomes for each target site were analyzed. The overall base editing efficiency for each target site was defined as:

$$\frac{{Total}\,{reads}\,{that}\,{contained}\,{one}\,{of}12\,{base}\,{transitions}\,{in}\,{target}\,{sites}}{{Total}\,{reads}}$$

Specifically, base editing frequency within the editing window was defined as:

$$\frac{{Total}\,{reads}\,{that}\,{contained}\,{one}\,{of}12\,{base}\,{transitions}\,{in}\,{window}\,{of}\,{target}\,{sites}}{{Total}\,{reads}}$$

The base editing frequency outside of the editing window was defined as:

$$\frac{{Total}\,{reads}\,{that}\,{contained}\,{one}\,{of}12\,{base}\,{transitions}\,{outside}\,{the}\,{window}\,{of}\,{target}\,{sites}}{{Total}\,{reads}}$$

Similarly, total reads were the sum of all aligned reads to the reference.

The purity frequency was calculated as:

$$\frac{{Total}\,{reads}\,{that}\,{contained}\,{one}\,{of}12\,{base}\,{transitions}\,{in}\,{target}\,{sites}}{{Total}\,{reads}\,{with}\,{mutations}}$$

The final relative editing frequency was normalized purity frequency so that the sum of relative editing frequency of 12 base transitions was 100%. The statistical position (overall sg, in the window, and outside the window) was the same as that of the base editing efficiency.

### Quantifying 1 bp indels within the protospacer of target site

To achieve accurate assessment for 1 bp indels, target sites were ordered by their indel frequency for each indel length, and the target sites with indel frequencies between the 25th and the 75th percentile were retained for following analysis^11^. Indel frequency was normalized by indel length, and the indel frequency at each position was calculated as averaged indel frequency of retained target sites.

The indel frequency for each target site was divided into deaminase-induced (position 1–11) and nCas9-induced (position 14–20) based on the location on the protospacer. The deaminase- or nCas9-induced indel frequency was calculated by the number of reads containing indels in the corresponding region divided by total number of reads.

### The ratio of base editing efficiency to indel frequency

Base editing efficiency to indel frequency ratios were calculated to uncover the relationship between general base editing efficiency and indel frequency. Target sites without indel reads were removed from this analysis to avoid division by zero. The geometric mean was selected as a summary statistic because BE:indel ratios were reported roughly by log-normalization, and the statistic summarizes more of the data than the median^11^.

### Sequence motif and logistic regression model

To clarify the preference of sequence context adjacent to targeted Cs for CBE or As for ABE within the editing window, 7 bp sequences (3 bp upstream of the targeted C/A, 1 bp targeted C/A and 3 bp downstream of the targeted C/A) for each targeted C/A were divided into training and test datasets with a ratio of 7:3. A logistic regression model was built on the training dataset using the sequence features, and tested on the test dataset. All sequence features surrounding the targeted C or A were encoded by one hot encoding. To illustrate whether integrated library and endogenous profiles share similar sequence features, training model built on integrated datasets was applied to predict non-zero base editing efficiency of endogenous datasets. The performance of logistic model was evaluated by Pearson correlation coefficient between the predicted and observed base editing efficiency.

### Characterizing base editing efficiency between endogenous and integrated target sites

Target sites were divided into three groups according to the differences in base editing efficiency between endogenous and integrated target sites. We fitted a linear regression of base editing efficiency for endogenous with that of integrated target sites, and the distribution of differences between the observed efficiency at endogenous sites and fitted ones was calculated and plotted. The $\mu \pm \sigma$ of difference distribution was considered as the cutoffs for categorization of target sites, where $\mu$ and $\sigma$ represent mean and standard deviation of the difference distribution, respectively. Target sites with differences higher than $\mu +\sigma$ were divided as Endo-bias group, and those with differences less than $\mu -\sigma$ were divided into Inte-bias group. The rest of target sites were divided into consistent group.

### Editing outcome analysis

For each target site, a strict prepossessing step was introduced for editing outcome analysis, 1) percentage of reads that were edited, which was defined as Ne/Nt, where Ne represents reads that were modified, and Nt represents total reads. 2) Kullback-Leibler divergence (KL) values of each target site among biological replicates. Target sites with Ne/Nt less than 90% and KL values extremely large (outliers) in each biological replicate were filtered out. Then the editing alleles of each target site from all the biological replicates were collapsed and proportions of editing outcomes were obtained for each target. For comparison of editing outcomes between endogenous and integrated target sites, the wild-type alleles were removed, and the left alleles were normalized to 100% for each target site.

Then the editing product allele forms with normalized proportion larger than 5% were kept. Target sites were assigned to four groups based on the consistency in outcome products between the Inte- and Endo- datasets. The “Consistent” group included target sites with the same outcome products between endogenous and integrated datasets. The “Inte-bias” group included target sites with outcome products from the Inte-datasets containing all the alleles from the Endo-datasets together with alleles forms unique to the Inte-datasets. The “Endo-bias” group represented target sites with outcome products from the Endo-datasets containing all the alleles from the Inte-datasets together with alleles forms unique to the Endo-datasets. The “Discordant” group represented those with editing outcome products unique to both Endo- and Inte-datasets.

### Quantification of transcriptional and epigenetic activity at target sites and their effects on editing efficiency

To reveal the effect of transcription activity and epigenetic modification on base editing efficiency of endogenous target sites, gene expression matrix from GEO database with accession GSE168012^23^, ChIA-PET assay narrow peak data of RNA polymerase II (Pol II)^21^ and CCCTC-binding factor (CTCF)^21^, DNase-seq^20^, and ChIP-seq narrow peak data of H3K4me1^21^, H3K4me3^20^, H3K27ac^21^ and H3K36me3^21^ from the Encyclopedia of DNA Elements (ENCODE), were retrieved. To quantify the level of endogenous factor at each target site, bedtools (version 2.27.1) were applied to map the location of each of the endogenous factors to the genomic loci of each target site. The gene activity for each target site was designated as the gene expression levels for the located gene in the genome. For gene expression levels, normalized gene expression levels larger and lower than 1.5 were assigned as “high” and “low” groups, respectively. Considering the long-range regulation of epigenetic factors on the genome, a wider range adjacent to the target site (65 bp upstream, 20 bp protospacer and 65 bp downstream of the protospacer for 150 bp target site and 500 bp upstream, 20 bp protospacer and 500 bp downstream of the protospacer for 1020 bp target site) for H3K27ac modification was considered. Target sites that overlapped with narrow peaks of histone modifications or broad peaks of CTCF/Pol II for one of the endogenous factors were considered with the corresponding modification.

Whole genome bisulfite sequencing (WGBS) data of HEK293T cells were obtained from the GEO database with accession GSE168012^23^. Downstream analysis was performed in R with BS-seq and DSS packages to obtain methylation levels for each single nucleotide. Then the per-base methylation level datas were intersected with the genomic position of target sites using bedtools (version 2.27.1). Target-level methylation was measured as average methylation levels of intersected nucleotides within the target site. Target sites with methylation levels larger than 0.75 were designated as high methylation group, and the others were labeled as non-methylation. To explore the base-level methylation at each base, we considered all targeted Cs for CBE and ACG/CGA motifs for ABE within the editing windows. The considered Cs or As that overlapped with the per-base methylation level data were classified as target sites with “Methylation”.

### Normalization of endogenous factors

Genome-wide signals of each endogenous factor were log_2_ transformed and normalized using min-max normalization method. For gene expression and DNase-seq data, Transcripts Per Million (TPM) and Reads Per Fragment (RPF) reads were used. For methylation data, beta value was used. For histone modification data, signal values (fold changes in comparison with the control) were used according to the Encode instruction.

To demonstrate the effect of endogenous factors on editing efficiencies within the editing window, the target sites were split into binary groups for each examined endogenous factor and compared the efficiency differences between two groups. Then regression models were built using continuous values of each endogenous factor and regressed out the number of As or Cs within the editing window.

### Quantification of methylation and histone modification on the editing efficiency was independent of motifs or not

To explore whether methylation and histone modification on the editing efficiency was independent of the underlying sequence motifs or not, we divided motifs into promoted motifs (including TAT, TAC, CAT, GAC, GAT) and inhibited motifs (including CAG, AAG, CAA, AAA). And then we calculated the ratio of promoted motifs as below in both non-methy and methy or H3K27ac and non-H3K27ac groups.

$$\frac{{Numbers}\,{of}\,{promoted}\,{motifs}}{{Numbers}\,{of}\,{promoted}\,{motifs}+{Numbers}\,{of}\,{inhibited}\,{motifs}}$$

The *P* value was calculated using χ2 test.

### Construction of base editing efficiency model

For predicting the editing efficiency of ABE and CBE at endogenous target sites, a deep-learning model based on Convolutional Neural Network (CNN) architecture was built. The model was consisted of 5 convolution layers with various kernel size (1 × 2, 1 × 3, 1 × 4, 1 × 5), one concatenate layer, one max pool layer (1 × 2) and one 20-size vector output layer. For BE_Seq efficiency model, the input layer was the 40 bp one-hot encoding matrix with 4 channels. For BE_Endo efficiency model, the input layer was 40 bp one-hot encoding matrix with 4 channels and additional one or more endo-factor channels. We adopted a common approach for deep neural network training, stratified the shuffled Endo-datasets based on the existence of endogenous factor into 80% (training set) and 20% (testing set), and repeated this process for six times to generate 6 shuffled datasets. During the training process, we randomly split 20% of the training dataset as validation set in every epoch for parameter optimization, and tested the model performance in the testing set. Finally, the performance of BE_Endo and BE_Seq efficiency models were evaluated using independent the MTSS, pathogenic datasets, Intergenic dataset and testing datasets of BE-HIVE, DeepBE and BE-DICT.

### Construction of proportional model

The proportional model was adapted from our previously published work^30^. Briefly, the editing of each position was considered as a Bernoulli distribution. In order to further output the proportion of all outcomes, a Markov network was introduced to model such dependency between each position. To simplify this problem, only the relation between adjacent editing positions was considered. Such Markov network was equivalent to a Bayesian network. We first proved that this simplification is reasonable by calculating the mutual information (log_2_ transformed) when the adjacent editing positions were considered or not. The majority of the information between distant editing positions was accounted for by joint probability with the adjacent editing position. Thus, this model not only considered the relation between adjacent editing positions, but also considered the relation between distant editing positions in the proportion model. The correlation of distant editing positions for each target site was transmitted through the chain-like Bayesian network. The probability of each position being edited can be obtained from the neuronal network model above. The correlation between different editing position was estimated using $c=\frac{{p}_{11}{p}_{00}}{{p}_{01}{p}_{10}}$ from the training dataset. Here ${p}_{11}$ and ${p}_{00}$ denoted the two positions being edited or not simultaneously, and ${p}_{01}$ and ${p}_{10}$ denoted the two positions being edited separately.

The above learning process can be formulated as follows. For a sequence$s$, the editing positions were denoted as ${X}_{1},{X}_{2},..{X}_{n}$. The joint probability of the Bayesian network was defined as $p\left({X}_{1},{X}_{2},\ldots ,{X}_{n}\right)=p\left({X}_{1}\right)p\left({X}_{2},|,{X}_{1}\right)\cdots p\left({X}_{n-1},|,{X}_{n}\right)$. The editing efficiency $p\left({X}_{i}=1\right)$ of each position was estimated by the output of the neuronal network $g\left(s,{X}_{i}\right)$, for $i=\mathrm{1,2},\ldots n$. The conditional probability $p\left({X}_{i},|,{X}_{i-1}\right)$ can be learned using $g\left(s,{X}_{i-1}\right),g\left(s,{X}_{i}\right)$ and the correlation $c$ between position ${X}_{i},{X}_{i-1}$. The proportions of all outcomes can be then inferred from the Bayesian network. The BE_Endo proportion was established from Endo-datasets (training and testing datasets) and validated independently using MTSS and pathogenic datasets. The proportions of Max-form of each target site were compared between predicted and measured values by both BE_Seq and BE_Endo proportion models.

### Comparison of different models

To compare the performance of several computational models for ABE and CBE, we implemented three BE tools using deep learning and machine learning methods, including DeepBE (https://github.com/MyungjaeSong/Paired-Library/tree/DeepCRISPR.info/DeepBaseEditor), BE-DICT (https://github.com/uzh-dqbm-cmi/crispr) and BE-HIVE (https://github.com/maxwshen/ be_predict_bystander).

DeepBE^9^, a deep learning predictor based on CNN architecture, were implemented by python 2.7 and TensorFlow 1.4. Each target sequence contains 26 bp, including 3 bp flanking + 20 bp protospacer + 3 bp PAM. A 2-step-implementation was required for DeepBE. Firstly, the editing efficiencies of each target sequence were predicted. Secondly, the proportions were predicted.

BE-DICT^13^, which was adapting the Transformer architecture, was built by a machine learning algorithm capable of predicting base editing outcomes of commonly used ABEs and CBEs at any given protospacer sequences. The ABEmax model for ABE and BE4max model for were chosen for further analysis. The inputs of target sequences only contain 20 bp protospacer and the outputs were the predicted score for each mutation variant under each ID of the sequences.

BE-HIVE^11^, a deep conditional autoregressive model, with two neural networks for encoding and decoding, was implemented by python package pyTorch 1.1 and python 3.7. For BE-HIVE, the inputs of sequences were expanded to 50 bp, containing 20 bp flanking + 20 bp protospacer + 3 bp PAM + 7 bp flanking sequence. The outputs contain the mutations that occurred out of the protospacer. Therefore, we calculated the sum of variation probabilities by considering all identical variants within the protospacers as homogenous variants and ignoring the mutations in the flanking sequences.

Firstly, we compared the prediction accuracy of BE_Endo with DeepBE, BE-DICT and BE-HIVE using testing datasets from the corresponding studies. Secondly, the performance of DeepBE, BE-DICT, BE-HIVE and BE_Endo was also evaluated using Endo, MTSS and Pathogenic datasets from this study. We concatenated all predicted outputs by the same sequence id and mutation variant with the experimental results. Pearson’s *R* was calculated between the observed experimental results and the predicted results of each model. Error bars demonstrated 95% confidence intervals. *P* values were calculated by two-sided Steiger’s Z tests.

### Statistical analysis

To compare base editing between endogenous and integrated protospacers, Pearson correlation coefficient was used to evaluate similarity. Wilcoxon rank-sum test was used to compute the significance of differences between groups. χ2 test was applied to test significance of epigenetic factors for any two groups of three divided groups and the promoted motif ratio (promoted motif number/ (promoted motif number + inhibited motif number)) for methylation and non-methylation group or H3K27ac and non-H3K27ac group of ABE, respectively. Pearson correlation coefficient (*R*) was calculated to evaluate the performance of each model. All the statistical tests were performed in R (version 4.1.2) and Python (version 3.9.4).

### Supplementary information

Supplementary Information

Supplementary Table S1

Supplementary Table S2

## Data Availability

All the sequencing data were deposited in the NCBI Sequence Read Archive (SRA) under project accession no. PRJNA885970. All scripts used in this study could be assessed through https://github.com/zhaodalv/BE_Endo.
