# Supplementary material for: Deep learning models incorporating endogenous factors beyond DNA sequences improve the prediction accuracy of base editing outcomes
Source: Cell Discov. 2024 Feb 20;10:20. doi: 10.1038/s41421-023-00624-1 (PMC10879117; doi:10.1038/s41421-023-00624-1)
Supplement: Supplementary file 1 — Supplementary Information [file 41421_2023_624_MOESM1_ESM.pdf]

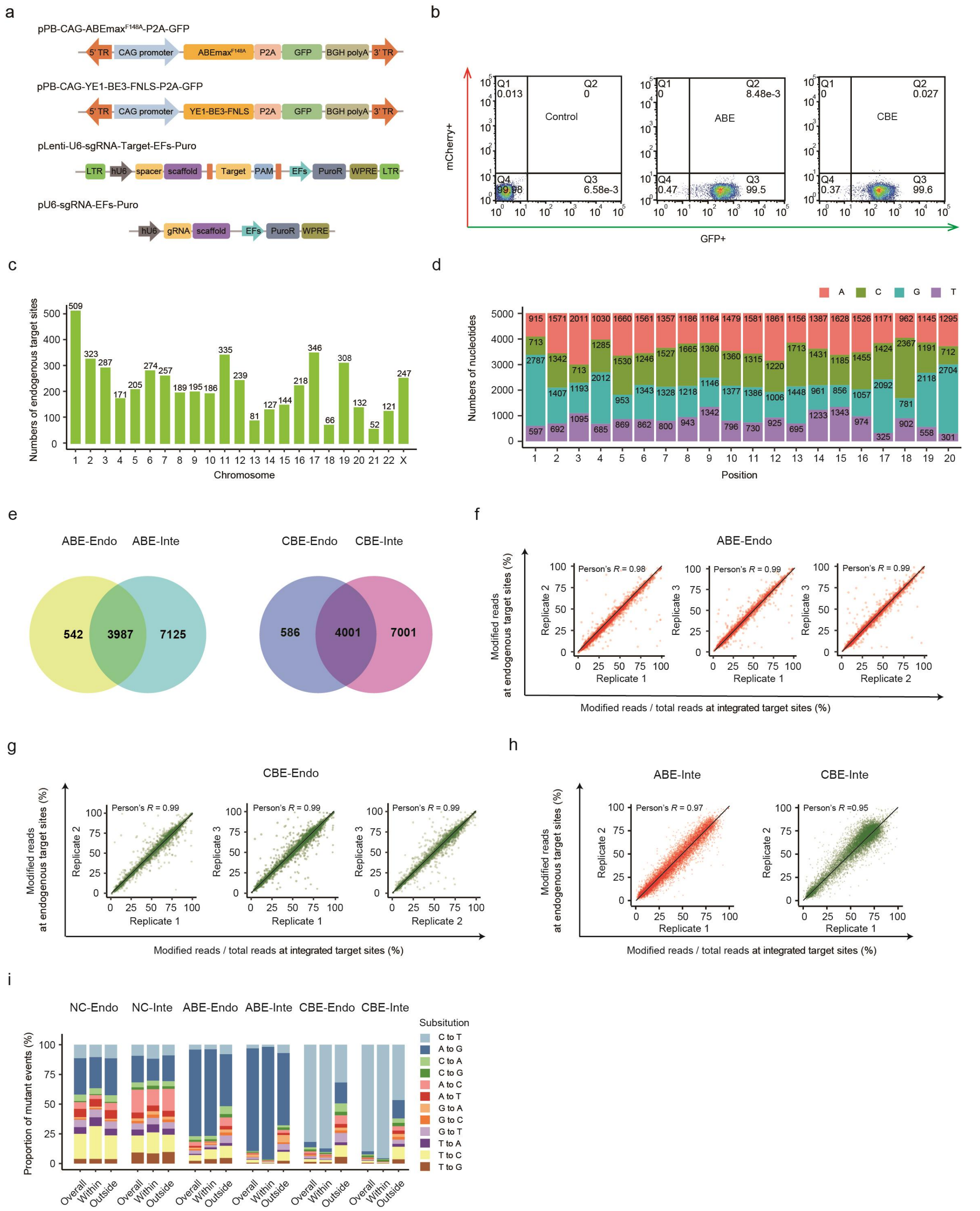

Supplementary Fig. S1. Generation of genome-wide Endo- and Inte-datasets.

a Schematic of BEs, paired lentiviral plasmid libraries, and sgRNA plasmids. **b** Gating strategy to separate GFP negative (control) and positive (ABE or CBE integrated) cells. **c** Chromosomal distribution of endogenous target sites across the genome. **d** Statistical numbers of 4 nt at each protospacer position 1–20 (PAM is at positions 21–23) of target sites. **e** Overlap of the target sites detected in ABE-Endo and ABE-Inte datasets (left) or CBE-Endo and CBE-Inte (right) datasets. **f-h** Correlation of editing efficiencies between biological replicates of ABE-Endo (**f**), CBE-Endo (**g**), ABE-Inte (**h**), and CBE-Inte (**h**) datasets. n = 4492 (replication 1 and 2), 4499 (replication 1 and 3) and 4484 (replication 2 and 3) for ABE-Endo. n = 4515 (replication 1 and 2), 520 (replication 1

and 3) and 4520 (replication 2 and 3) for CBE-Endo. n = 11112 (replication 1 and 2) for ABE-Inte. n = 11002 (replication 1 and 2) for CBE-Inte. **i** Relative proportions of 12 types of base substitutions within the Endo- and Inte- datasets.

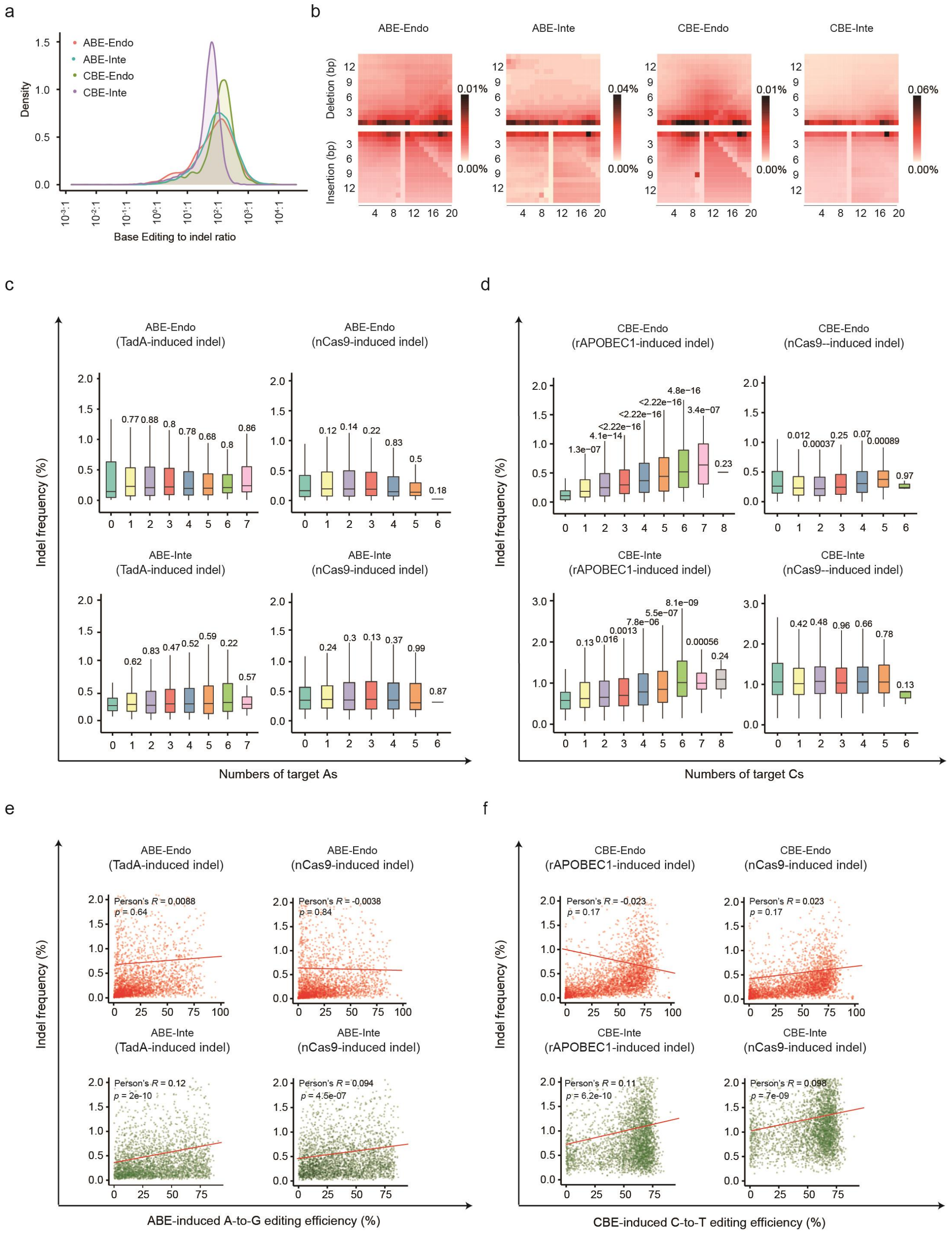

Supplementary Fig. S2. Comparison of ABE or CBE-directed indel frequency at endogenous and integrated target sites.

a Distribution of base editing: indel ratio values at endogenous and integrated target sites. **b** Heatmap of indel frequency among edited reads by position and length. **c** Correlation between deaminase TadA-induced (left) or nCas9-induced (right) indel frequencies and number of targeted As within each protospacer in the ABE-Endo and ABE-Inte datasets. n = 67 (0), 379 (1), 911 (2), 1126 (3), 874 (4), 444 (5), 142 (6) and 15 (7) for ABE-Endo TadA. n = 66 (0), 369 (1), 900 (2), 1086 (3), 838 (4), 418 (5), 136 (6) and14 (7) for ABE-Inte TadA. n = 427 (0), 1189(1), 1291 (2),

757 (3), 236 (4), 37 (5) and 1 (6) for ABE-Endo nCas9. *n* =431 (0), 1185 (1), 1279 (2), 741 (3), 233 (4), 34 (5) and 1 (6) for ABE-Inte nCas9. **d** Correlation between deaminase rAPOBEC1-induced (left) or nCas9-induced (right) indel frequencies and number of targeted Cs within each protospacer in the CBE-Endo and CBE-Inte datasets. *n* = 121 (0), 554 (1), 1046 (2), 1072 (3), 739 (4), 338 (5), 84 (6), 18 (7) and 2 (8) for CBE-Endo rAPOBEC1. *n* = 123 (0), 556 (1), 1046 (2), 1066 (3), 739 (4), 338 (5), 84 (6), 18 (7) and 2 (8) for CBE-Inte rAPOBEC1. *n* = 417 (0), 1068 (1), 1274 (2), 811 (3), 330 (4), 67 (5) and 1 (6) for CBE-Endo nCas9. *n* = 416 (0), 1075 (1), 1280 (2), 819 (3), 331 (4), 67 (5) and 1 (6) for CBE-Inte nCas9. **e** Correlation of ABE-directed A-to-G editing efficiency and deaminase TadA-induced or nCas9-induced indel frequencies at endogenous (upper) and integrated (down) target sites. *n* = 2910 (ABE-Endo TadA), 2819 (ABE-Inte TadA), 2899 (ABE-Endo nCas9) and 3879 (ABE-Inte nCas9). **f** Correlation of CBE-directed C-to-T editing efficiency and deaminase rAPOBEC1-induced or nCas9-induced indel frequencies at endogenous (upper) and integrated (down) target sites. *n* = 3464 (CBE-Endo rAPOBEC1), 3450 (CBE-Inte rAPOBEC1), 3461 (CBE-Endo nCas9) and 2463 (CBE-Inte nCas9).

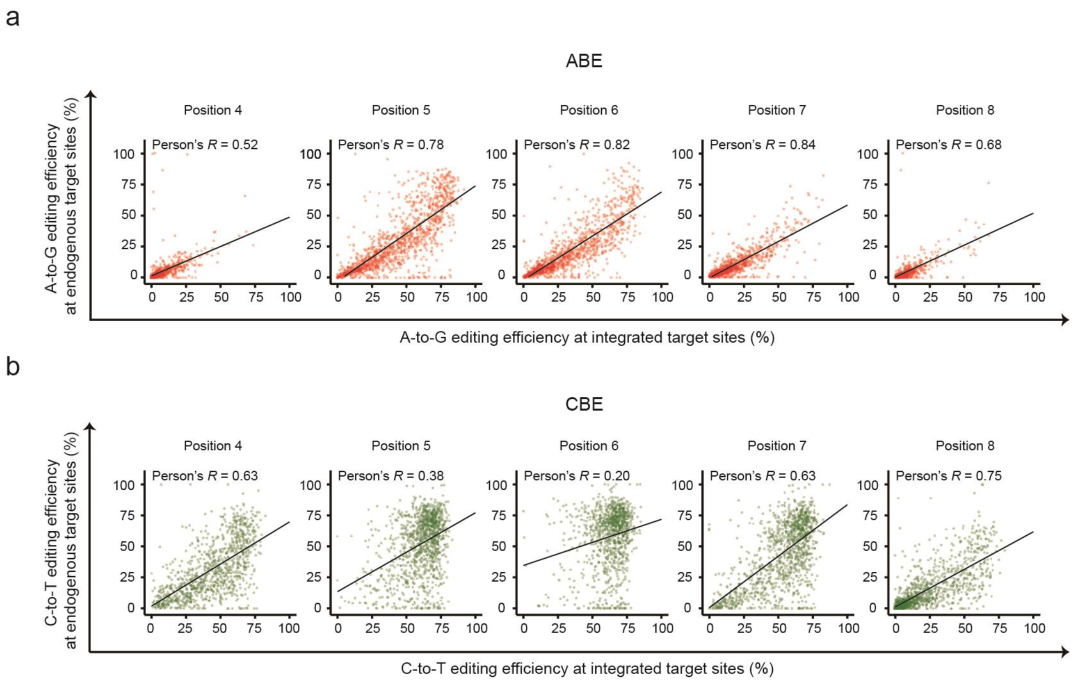

Supplementary Fig. S3. Correlation of base editing efficiency between the Endo- and Inte-datasets.

a Correlation of ABE-induced A-to-G base editing efficiency between Endo-and Inte-datasets at protospacer positions 4–8. n = 809 (position 4), 1343, 1250, 1106 and 916 (position 8). **b** Correlation of CBE-induced C-to-T base editing efficiency between Endo- and Inte-datasets at protospacer positions 4–8. n = 1025 (position 4), 1181, 1040, 1195 and 1343 (position 8).

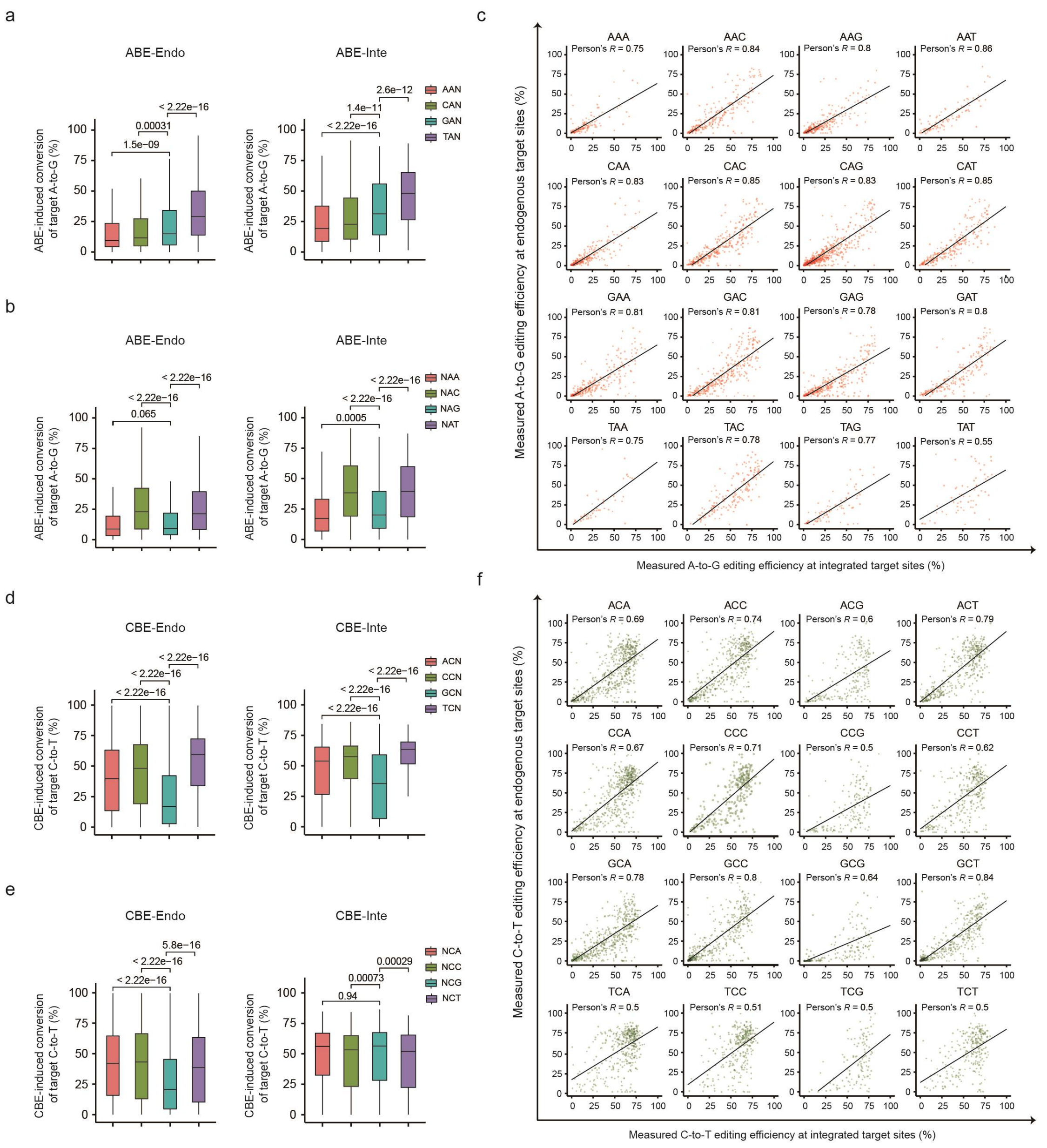

Supplementary Fig. S4. Effect of sequence context around the target sites on base editing efficiency within the editing window of ABE or CBE.

a A-to-G editing efficiency directed by ABE at endogenous and integrated target sites of As bearing different nucleotides upstream. N = A, T, G, or C. *P* values above each group were calculated in comparison with the "GAN" group. *n* = 762 (AAN), 1400 (CAN), 1163 (GAN) and 374 (TAN) for ABE. **b** A-to-G editing efficiency directed by ABE at endogenous and integrated target sites of As bearing different nucleotides downstream. N = A, T, G, or C. *P* values above each group were calculated in comparison with the "NAG" group. *n* = 732 (NAA), 1087 (NAC), 1270 (NAG) and 610 (NAT) for ABE. **c** Correlation of ABE-directed A-to-G editing efficiency at each indicated motif between endogenous and integrated datasets. *n* = 139 (AAA), 250 (AAC), 273 (AAG), 100 (AAT), 255 (CAA), 325 (CAC), 575 (CAG), 245 (CAT), 286 (GAA), 334 (GAC), 338 (GAG), 205 (GAT), 52 (TAA), 178 (TAC), 84 (TAG) and 60 (TAT) for ABE. **d** C-to-T editing

efficiency directed by CBE at endogenous and integrated target sites of Cs bearing different nucleotides upstream. N = A, T, G, or C. *P* values above each group were calculated in comparison with the "GCN" group. *n* = 1671 (ACN), 1308 (CCN), 1686 (GCN) and 1119 (TCN) for CBE. **e** C-to-T editing efficiency directed by CBE at endogenous and integrated target sites of Cs bearing different nucleotides downstream. N = A, T, G, or C. *P* values above each group were calculated in comparison with the "NCG" group. *n* = 2104 (NCA), 1528 (NCC), 682 (NCG) and 1470 (NCT) for CBE. **f** Correlation of CBE-directed C-to-T editing efficiency at each indicated motif between endogenous and integrated datasets. *n* = 582 (ACA), 463 (ACC), 213 (ACG), 413 (ACT), 537 (CCA), 284 (CCC), 159 (CCG), 328 (CCT), 585 (GCA), 454 (GCC), 186 (GCG), 461 (GCT), 400 (TCA), 327 (TCC), 124 (TCG) and 268 (TCT) for CBE.

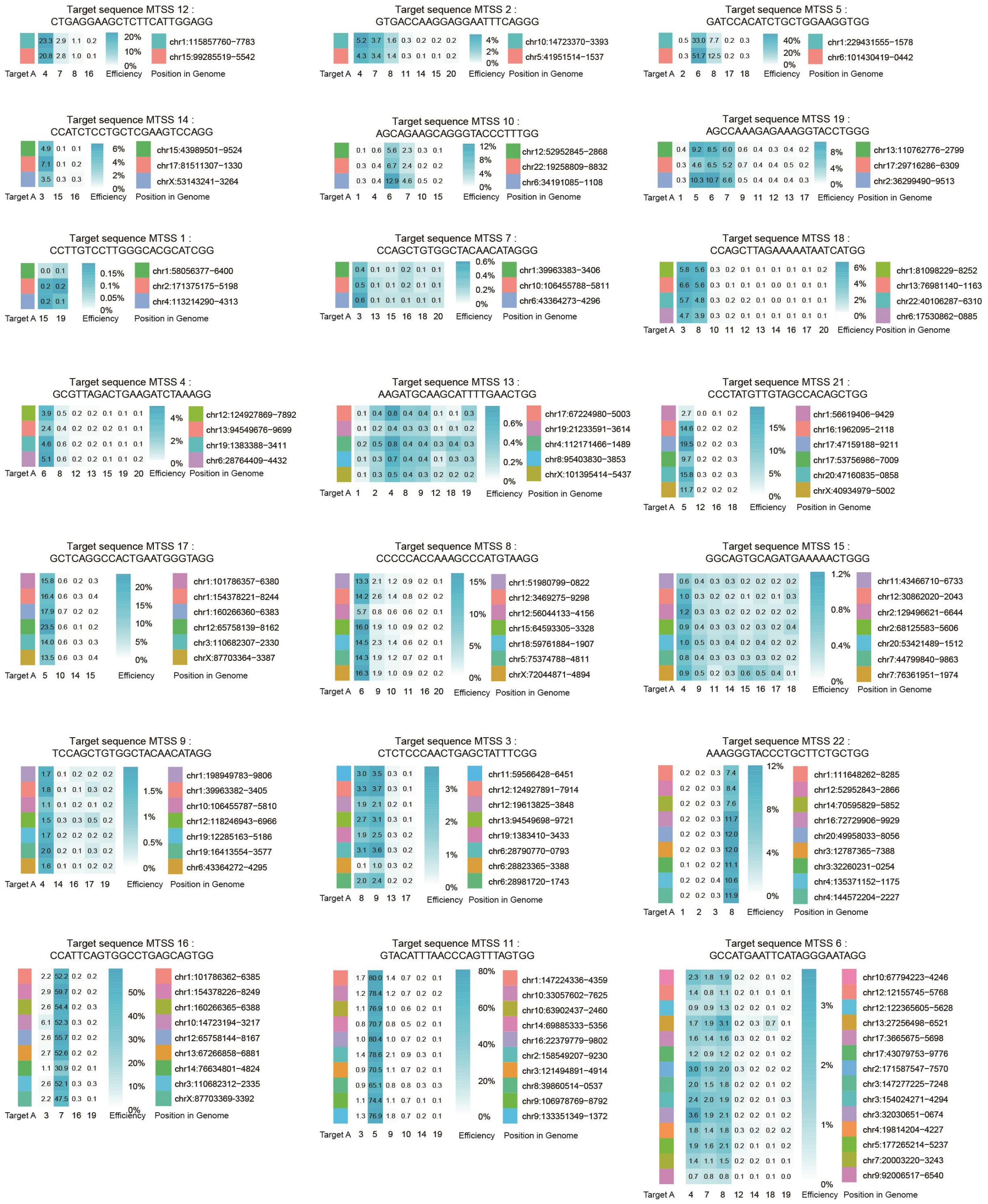

Supplementary Fig. S5. Characterization of ABE-directed A-to-G editing efficiency at 21 MTSS sites. Each target sequence occurs from 2 to 14 times within the genome. *n* = 3 biological replicates for each site.

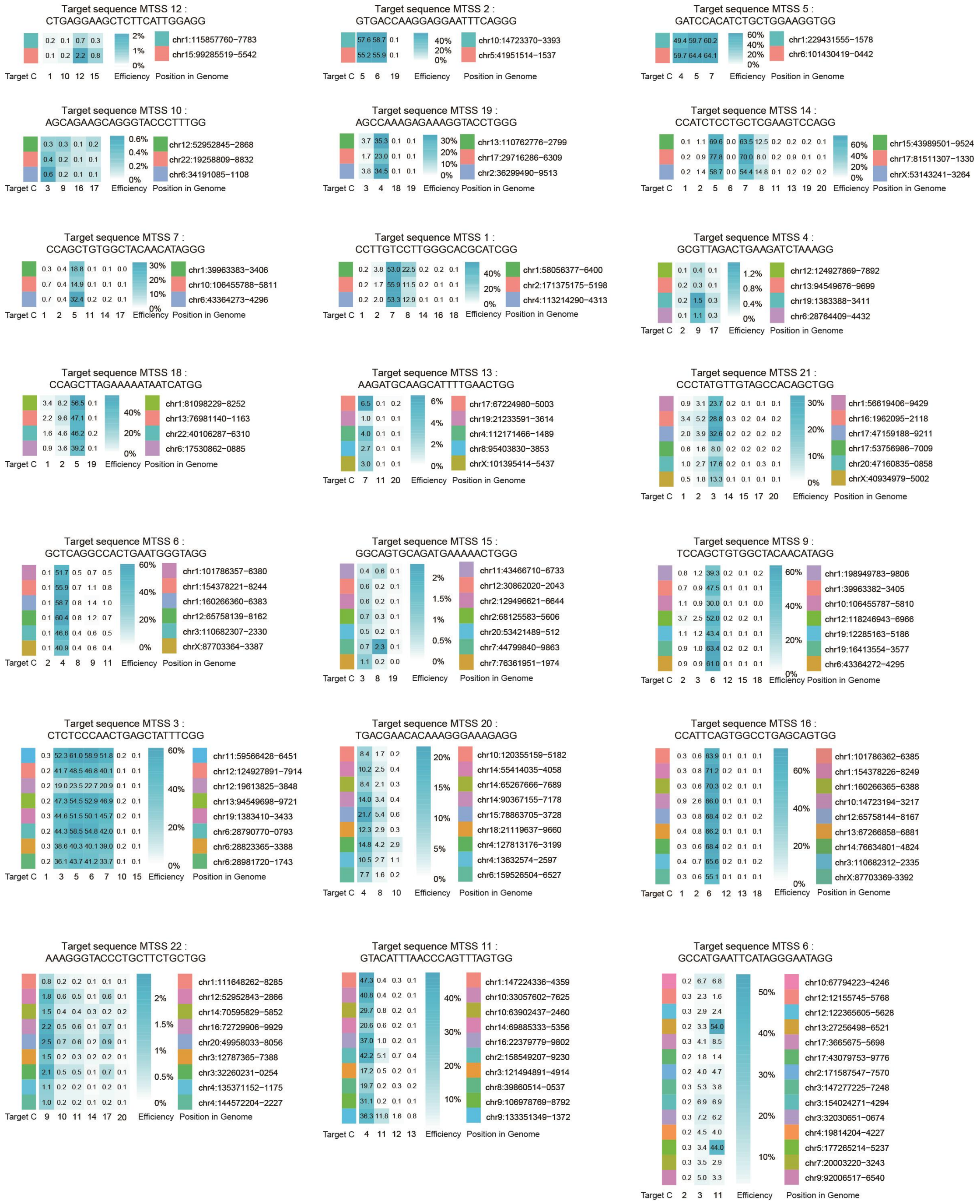

Supplementary Fig. S6. Characterization of CBE-directed C-to-T editing efficiency at 21 MTSS sites. Each target sequence occurs from 2 to 14 times within the genome. n = 3 biological replicates for each site.

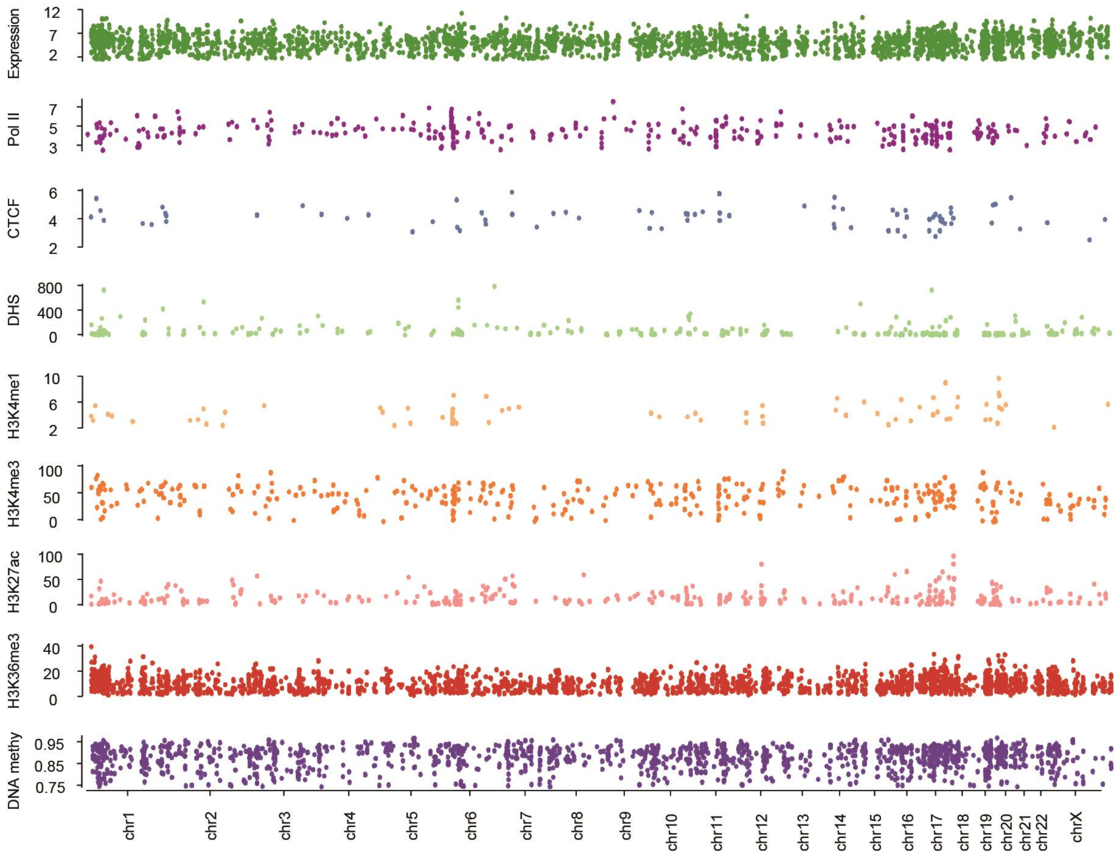

Supplementary Fig. S7. Distribution of the levels of indicated endogenous factors across the genome. n = 3078 (Expression), 376 (Pol II), 85 (CTCF), 307 (DHS), 82 (H3K4me1), 401 (H3K4me3), 375 (H3K27ac), 2194 (H3K36me3) and 1423 (DNA methy).

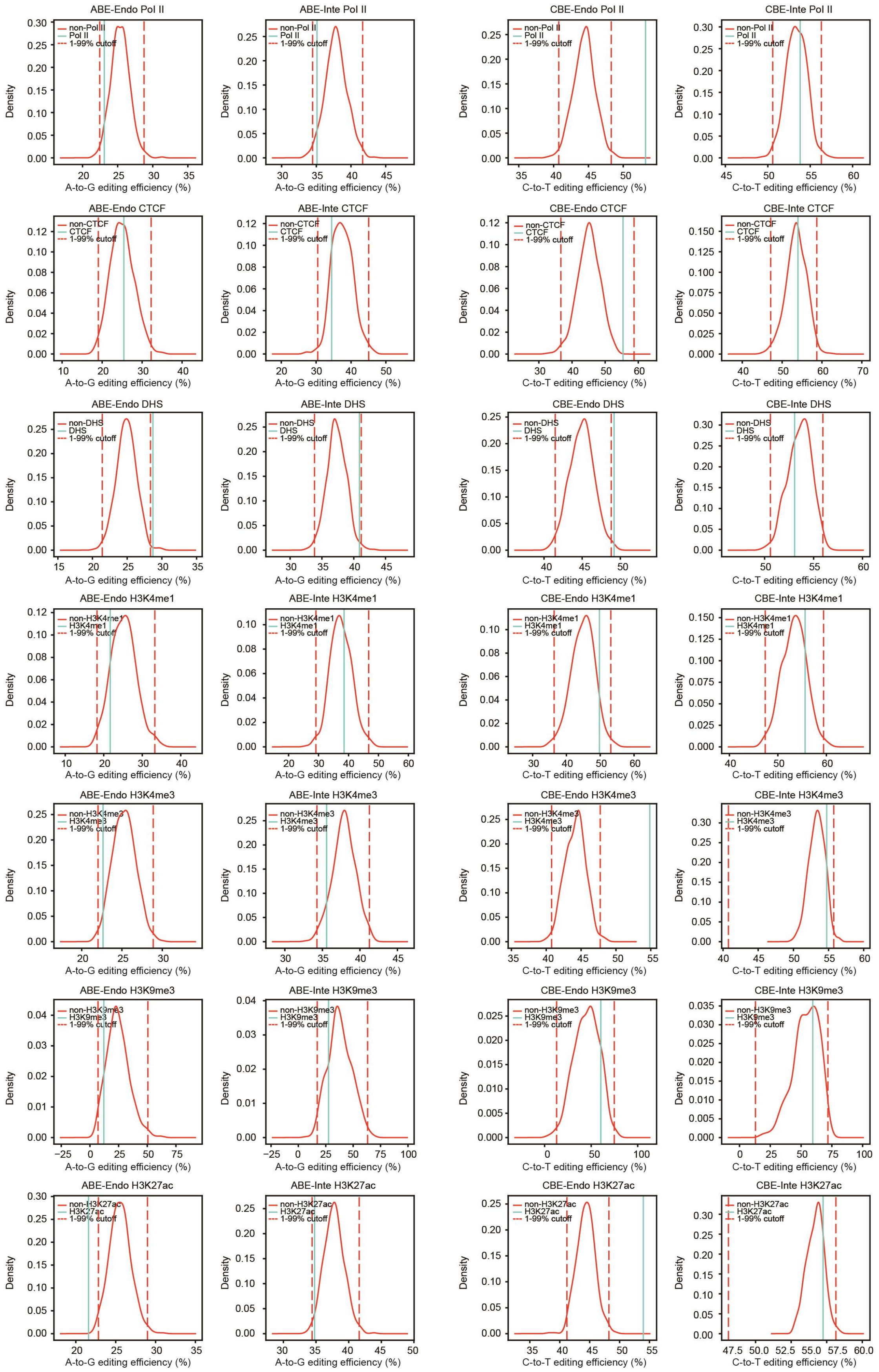

Supplementary Fig. S8. Comparison of BEs-directed base editing efficiency at endogenous target sites with or without endogenous factors. Distribution of BEs-directed base editing

efficiency at endogenous target sites without endogenous factors vs. with endogenous factors. The red dashed line represents the editing efficiency at 1% and 99% level of the random sampling distribution, while the green line indicates the average editing efficiency for target sites with the indicated endogenous factors.

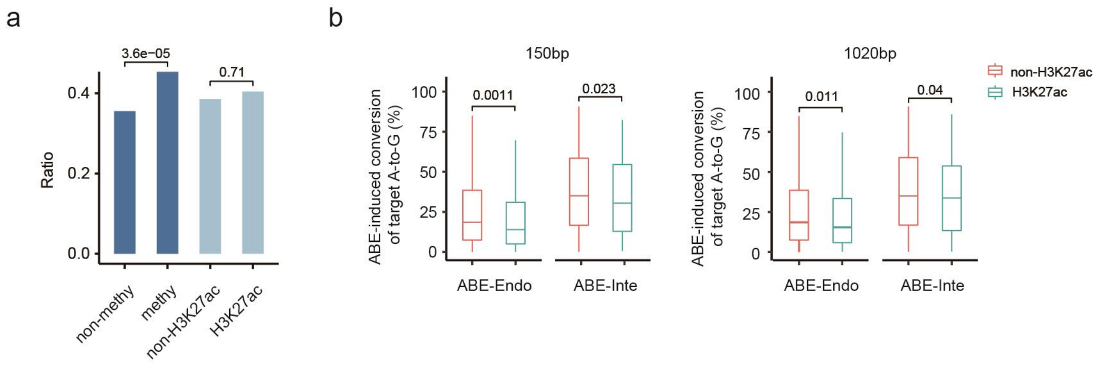

Supplementary Fig. S9. Effects of DNA methylation and H3K27ac modification on A-to-G editing efficiency within endogenous target sites.

a Comparison of the ratio of ABE preferred motifs at target sites with and without DNA methylation or H3K27ac modification. **b** Comparison of A-to-G editing efficiency at target sites with or without H3K27ac modification within 150 bp or 1,020 bp genomic regions adjacent to the target sites. n = 238 (H3K27ac) and 2668 (non-H3K27ac) for 150 bp. n = 329 (H3K27ac) and 2577 (non-H3K27ac) for 1020 bp.

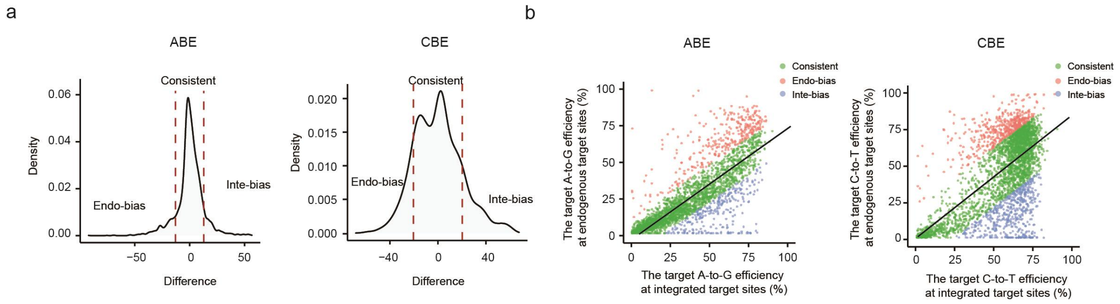

Supplementary Fig. S10. Classification of target sites in three groups based on the consistency of base editing efficiency values between endogenous and integrated datasets of ABE and CBE. **a** Distribution of the differences in editing efficiency between endogenous and integrated target sites. The red dashed lines represent the first and third quantiles of the differences. **b** Correlations of editing efficiency between endogenous and integrated target sites. The dot colors represent the three groups divided in (**a**). n = 2295 (Consistent), 333 (Endo-bias) and 278 (Inte-bias) for ABE, n = 2425 (Consistent), 504 (Endo-bias) and 521 (Inte-bias) for CBE,

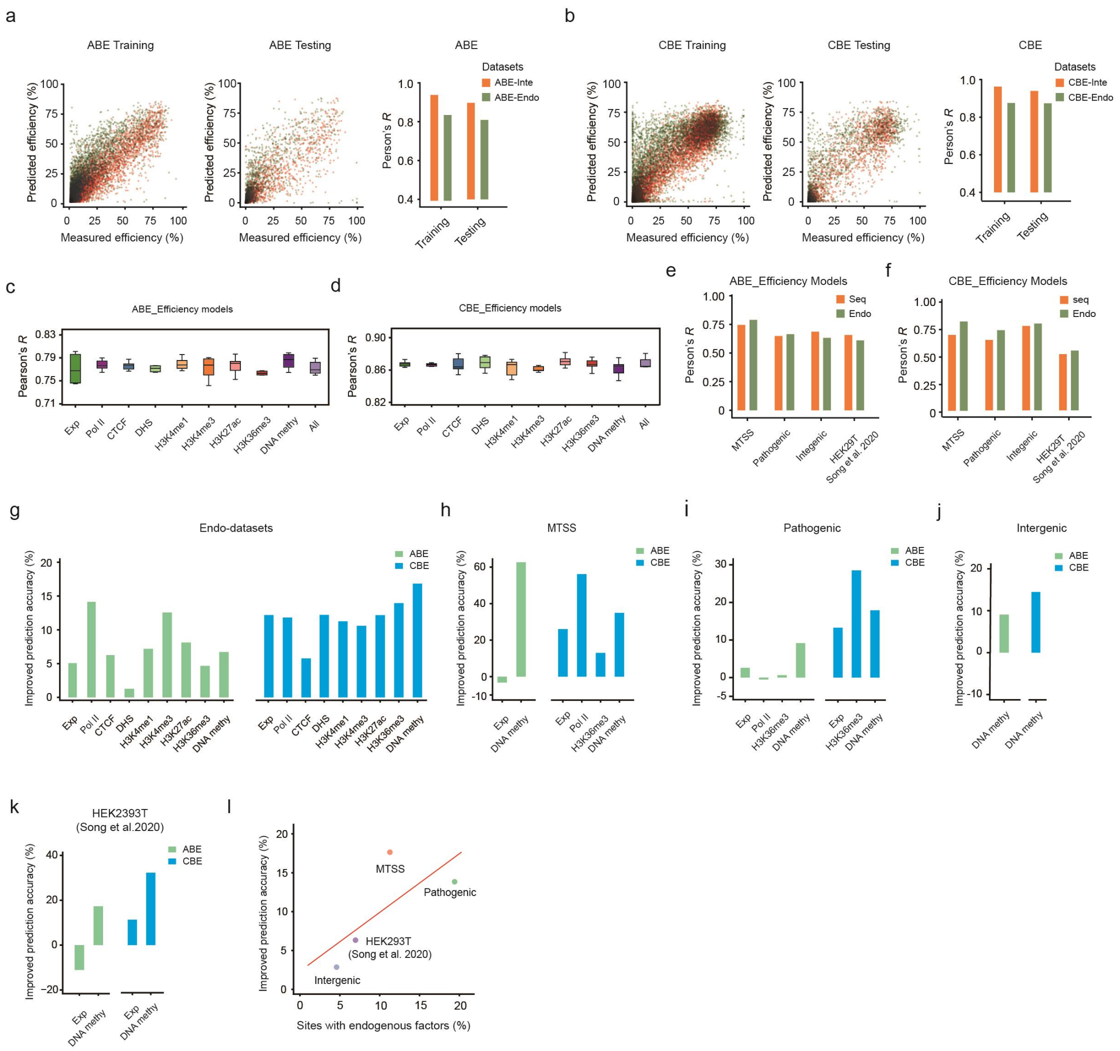

Supplementary Fig. S11. Performance evaluation of BEs efficiency models based on integrated and endogenous datasets.

a-**b** Performance evaluation of ABE (**a**) and CBE efficiency (**b**) models based on sequence features using integrated and corresponding endogenous datasets. **c** Performance evaluation of ABE efficiency models integrating one or all endogenous factors. n = 6 for each model. **d** Performance evaluation of CBE efficiency models integrating one or all endogenous factors. n = 6 for each model. **e** Performance evaluation of ABE_Seq and ABE_Endo efficiency models for predicting editing efficiency of target As within editing window using MTSS, Pathogenic, Intergenic and HEK293T (Song et al. 2020) datasets. **f** Performance evaluation of CBE_Seq and CBE_Endo efficiency models for predicting editing efficiency of target Cs within editing window using MTSS, Pathogenic, Intergenic and HEK293T (Song et al. 2020) datasets. **g-k** Performance evaluation of BEs efficiency models at target sites with different endogenous factors using Endo-datasets (**g**) and other 4 independent datasets, including MTSS (**h**), Pathogenic (**i**), Intergenic (**j**) and HEK293T (**k**) (Song et al. 2020) datasets. **l** Correlation between the proportions of targets with endogenous factors and improved prediction accuracy.

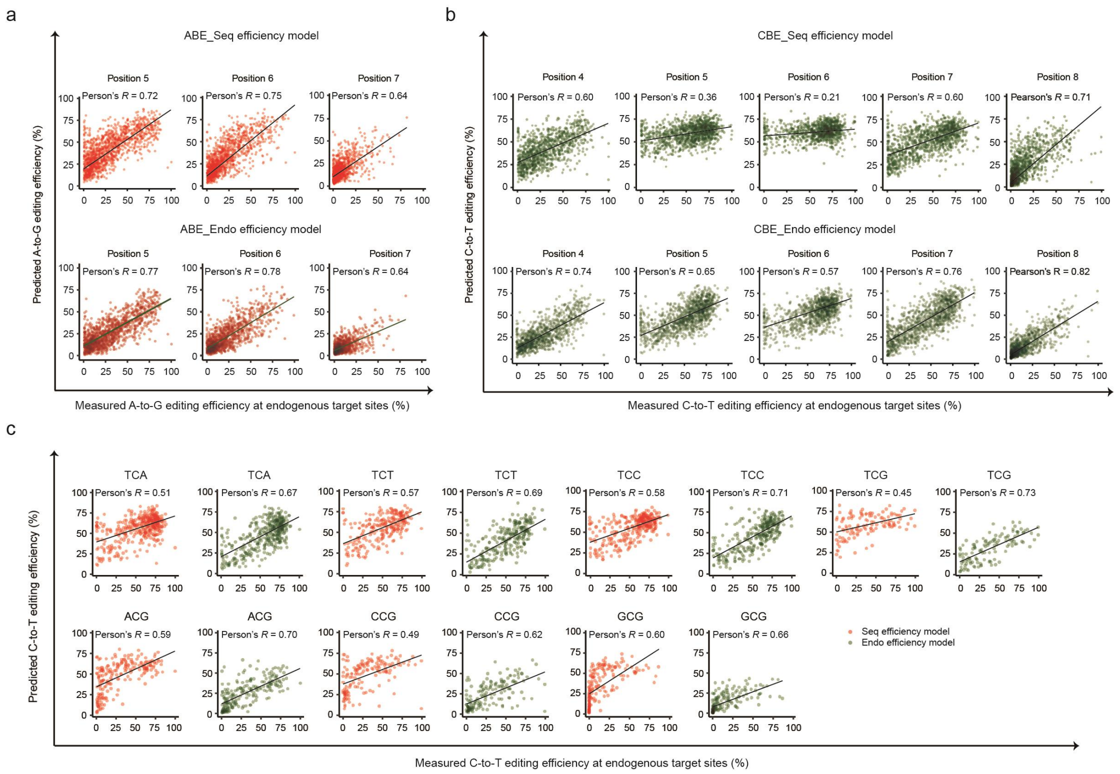

Supplementary Fig. S12. Performance evaluation of BEs efficiency models at endogenous target sites with indicated positions and motifs.

a Correlation between observed and predicted base editing efficiency from ABE_Seq and ABE_Endo efficiency models at positions 5-7. *n* = 1342 (position 5), 1248 and 1106 (position 7). **B** Correlation between observed and predicted base editing efficiency from CBE_Seq and CBE_Endo efficiency models at positions 4-8. *n* = 1025 (position 4), 1181, 1040, 1194 and 1343 (position 8). **c** Performance evaluation of CBE_Seq and CBE_Endo efficiency models at TCN and NCG motifs. *n* = 400 (TCA), 268 (778), 327 (TCC), 124 (TCG), 213 (ACG), 159 (CCG) and 186 (GCG).

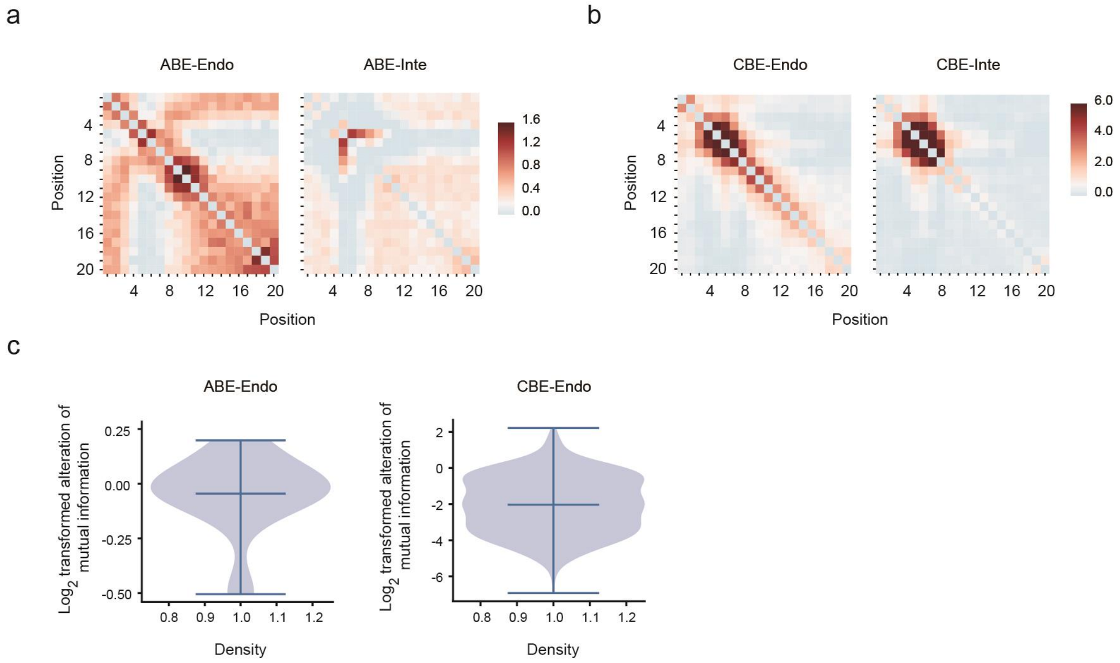

Supplementary Fig. S13. Co-occurrent editing between each pair of the target base.

a-b Prior information of the co-occurrent editing between each pair of the target nucleotides in the Endo- and Inte- datasets for ABE (**a**) and CBE (**b**). **c** Violin plot showing the alteration of mutual information (log_2 transformed) when the adjacent editing positions were considered or not in ABE and CBE.

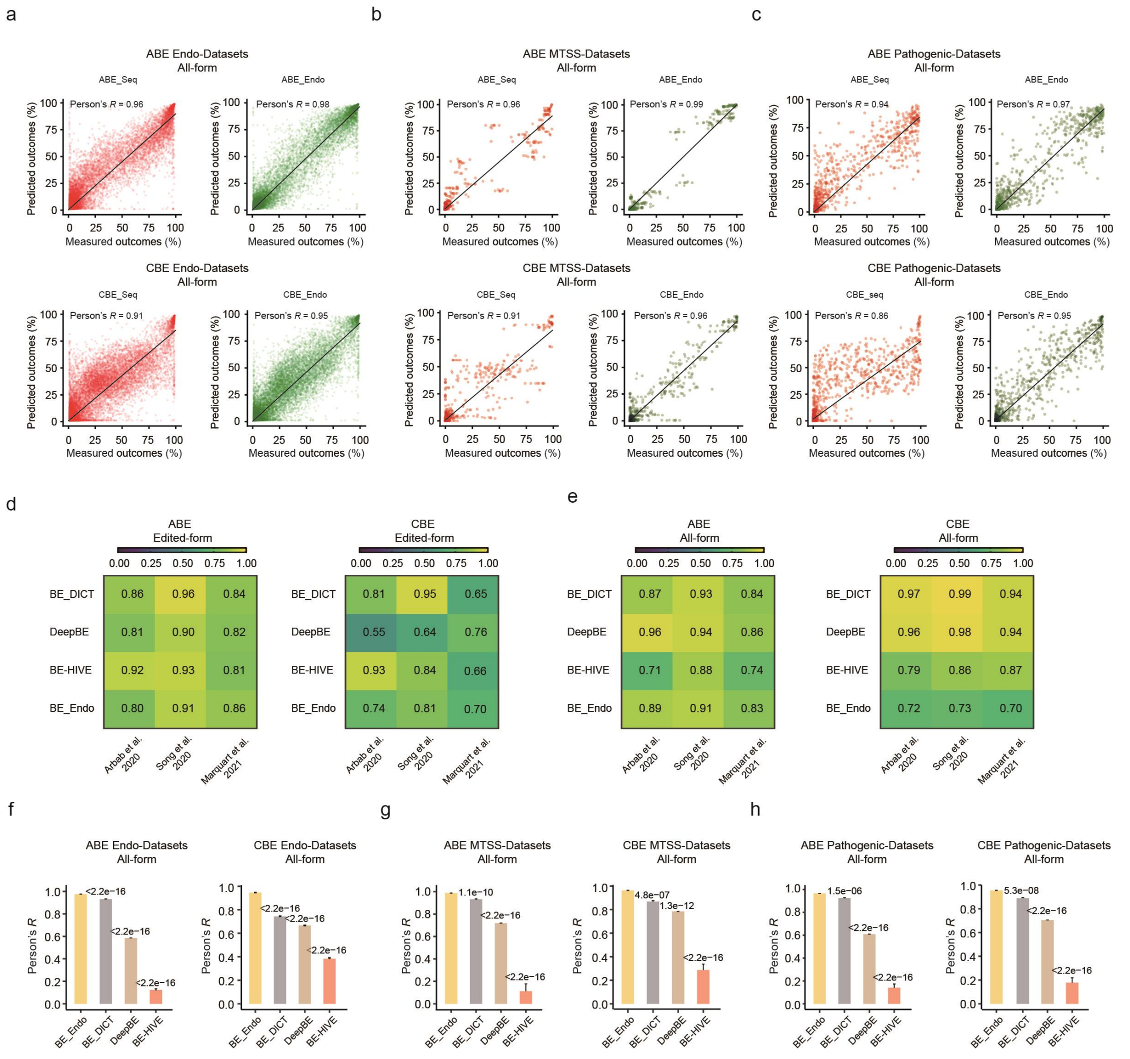

Supplementary Fig. S14. Performance evaluation of deep learning models for prediction of editing outcomes of BEs.

a-c Performance evaluation of BE_Seq and BE_Endo models for predicting all-form of edits using testing endogenous (**a**), independent MTSS (**b**) and Pathogenic (**c**) datasets. **d-e** Performance evaluation of different machine learning models of ABE and CBE in predicting edited (**d**) or all (**e**) outcomes using different integrated testing datasets. Pearson's R was calculated by comparing measured and predicted outcomes in the previous published datasets by Arbab et al., Song et al., and Marquart et al. n = 7743 (Edited-form for ABE), 7537 (Edited-form for CBE), 9008 (All-form for ABE) and 8895 (All-form for CBE) in the dataset from Arbab et al. n = 1754 (Edited-form for ABE), 2316 (Edited-form for CBE), 2189 (All-form for ABE) and 2796 (All-form for CBE) in the dataset from Song et al. n = 3838 (Edited-form for ABE), 4496 (Edited-form for CBE), 5502 (All-form for ABE) and 6165 (All-form for CBE) in the dataset from Marquart et al. **f-h** Performance evaluation of different models for ABE and CBE on prediction of the all-form of outcomes using the 3 datasets from this study. n = 87147 (Endo), 2651 (MTSS) and 5478 (Pathogenic) for ABE

(All-form). *n* = 81590 (Endo), 2437 (MTSS) and 4113 (Pathogenic) for CBE (All-form). *P* values above each group were calculated in comparison with the “BE_Endo” models.

Title: Supplementary table S1

Description: Information of endogenous, integrated, MTSS, pathogenic and intergenic target sites. This file provides the basic information of endogenous (Sheet 1), integrated (Sheet 2), MTSS (Sheet 3), pathogenic (Sheet 4 for ABE and 5 for CBE) and intergenic (Sheet 6) target sites of BEs in this study, including spacer sequences, target positions, primers for PCR,

Title: Supplementary table S2

Description: Endogenous factors of each target site measured in ABE-Endo and CBE-Endo datasets. This file provides the information of endogenous factors of each target site measured in ABE-Endo (Sheet 1) and CBE-Endo (Sheet 1) datasets including expression, Pol II, CTCF, DHS, Histone modifications, DNA methylation.
